# Contamination-controlled upper gastrointestinal microbiota profiling reveals salivary-duodenal community types linked to opportunistic pathogen carriage and inflammation

**DOI:** 10.1080/19490976.2025.2539452

**Published:** 2025-08-01

**Authors:** Nina S. Schmidt, Elisabeth Dörner, Daniel Podlesny, Elisabeth Bohlhammer, Alena M. Bubeck, Hannah K. Ruple, Vivian Tetzlaff-Lelleck, Christian Sina, Herbert Schmidt, W. Florian Fricke

**Affiliations:** a Department of Microbiome Research and Applied Bioinformatics, University of Hohenheim, Stuttgart, Germany; b European Molecular Biology Laboratory (EMBL), Molecular Systems Biology Unit, Heidelberg, Germany; c Institute of Nutritional Medicine, University of Lübeck and University Medical Center Schleswig-Holstein, Lübeck, Germany; d Department of Food Microbiology and Hygiene, University of Hohenheim, Stuttgart, Germany; e Institute for Genome Sciences, University of Maryland School of Medicine, Baltimore, USA

**Keywords:** Saliva, duodenum, mouse microbiota, human microbiota, microbiota transfer, extraintestinal infections, dysbiosis, contamination, upper gastrointestinal tract microbiota, microbiota types

## Abstract

The upper gastrointestinal (uGI) microbiota has been implicated in infectious, metabolic, and immunological conditions, yet remains poorly characterized due to invasive sampling and low microbial biomass. We developed and validated a contamination-controlled 16S rRNA gene and transcript-based protocol to profile the murine and human uGI microbiota from low-biomass samples. We applied this protocol to murine esophageal, gastric, and duodenal tissues, and to human saliva, gastric, and duodenal aspirates from patients undergoing endoscopy for suspected food-related, mild GI symptoms. Our objective was to identify conserved compositional and structural uGI microbiota patterns and assess their clinical relevance in relation to pathogen burden and inflammation. In mice, we found evidence for transcriptionally inactive and active intestinal taxa along the uGI tract, supporting horizontal microbiota transfer. In humans, we identified two distinct, inversely correlated salivary microbiota types – one dominated by the *Prevotella 7* genus – which were conserved in the duodenum. The *Prevotella 7*-dominated uGI microbiota type was associated with lower relative abundances of gastrointestinal and extraintestinal opportunistic pathogens. These patterns were reproducible in an independent cohort and associated with lower systemic TNF-α levels. Our findings suggest that noninvasive salivary microbiota profiling can stratify individuals based on uGI microbiota composition and inflammation-associated risk traits, offering new opportunities for clinical applications and translational studies.

## Introduction

The microbiota of the upper gastrointestinal (uGI) tract can affect the risk for and severity of diseases of the oral cavity,^1^ esophagus^2^ and stomach.^3,4^ In addition, commensal members of the uGI tract microbiota have been associated with the pathogenesis or exacerbation of intestinal disorders,^5,6^ as well nasal,^7^ respiratory^8^ and other extraintestinal infections,^9,10^ demonstrating their potential to act as opportunistic pathogens after translation to other body sites. Interference with the gastric milieu, via pharmaceutical (e.g. gastric acid-reducing proton pump inhibitors^11^ or surgical interventions (e.g. weight loss-inducing sleeve gastrectomy and Roux-en-Y gastric bypass surgery), modify taxonomic and functional traits of the intestinal microbiome, which can manifest with altered metabolism, reduced weight gain, and improved insulin sensitivity in ex-germ free mice colonized with fecal samples of treated patients.^12^ The stomach may therefore act as a modulator for intestinal microbiome composition and function, e.g. by controlling oral-intestinal transmission of ingested microbes from food and the oropharynx.^13–15^ Downstream of the stomach, the small intestine plays a crucial role in food digestion, nutrient absorption, and immune function and has been implicated in food allergies and intolerances.^16–20^ A better understanding of the microbial population structures and their interdependencies across different locations of the uGI tract could therefore help identify microbiota contributions to human health and disease.

The human uGI tract microbiome, especially of the stomach and small intestine, remains understudied. Sample collection typically relies on invasive procedures like esophagogastroduodenoscopy (EGD), which is clinically indicated only for patients with gastrointestinal symptoms, providing limited insights into microbiome organization and function under healthy, homeostatic conditions. Saliva, which can be sampled non-invasively, represents a more easily accessible sample type and has been suggested to reflect clinically relevant microbiome traits of the small intestine, for example in the context of small intestinal bacterial overgrowth (SIBO).^19^ By serving as a reservoir for opportunistic pathogens, the oral microbiota may also contribute to other GI and systemic diseases, including inflammatory bowel disease (IBD), arthritis, colorectal and pancreatic cancers, and Alzheimer’s disease.^21^ Provided that stable and consistent interdependencies exist between oral, gastric and small intestinal microbiomes, salivary microbiota analysis could therefore offer a window into lower GI microbiome structures and uncover diagnostic biomarkers and therapeutic targets for clinical applications.

Mice are widely used as an animal model to study structural and functional features of the human gut microbiome and, after transfer of human microbiome samples into germ-free mice, to demonstrate causality of microbiome-disease associations.^22–25^ The murine uGI tract shows anatomical and physiological differences to humans, most notably the non-glandular forestomach that serves as a food reservoir,^23,26^ and mice are coprophagic, which can affect gut microbiota composition and exchange.^22^ However, access to surgically obtained tissue samples from mice avoids the potential confounding of distinct uGI tract microbiomes in endoscopically collected human samples.^27,28^ Studying the microbiomes of low-microbial biomass samples, including from the uGI tract, remains a common, insufficiently recognized and experimentally addressed challenge,^29^ as low microbial densities can inflate contamination signals from extraction and amplification procedures, reagents and lab environments,^30^ and affect the biological interpretation of the resulting data.^27,28^ Mice can serve as a valuable model organism to develop and validate robust, contamination-controlled protocols for human uGI microbiota analysis and to investigate shared, general principles of uGI microbiota organization, including changes in microbiota composition and activity during oral-duodenal passage.^15^

In order to establish a protocol for uGI microbiota analysis based on a careful experimental and analytical characterization of contaminants, we compared esophagus, stomach and duodenum tissue samples from mice, along with an extensive set of controls in order to assess the influence of contamination introduced during each step of the processing and analysis protocol of these low-microbial biomass samples. We then applied the same validated protocol to investigate the relationship between the human oral, gastric, and duodenal microbiota and to study uGI microbiota structures in an adult population with relatively mild clinical symptoms due to suspected non-classical food intolerance. We describe evidence in the murine uGI microbiota composition and activity for horizontal microbiota transfer between mice, and identify novel organizational features of the human uGI microbiota, including the stratification of our cohort in microbiota types based on cumulative relative abundances of two dominant bacterial clusters, which are retained from saliva into the duodenum. Importantly, the same salivary-duodenal uGI microbiota typing could be reproduced in a separate, independent patient cohort and was consistently associated with clinically relevant parameters, including opportunistic pathogen burdens and systemic inflammation. This approach offers a blueprint for other low-microbial biomass microbiome projects and suggests opportunities for salivary microbiota-based patient stratification and clinical infectious risk assessment.

## Results

### The microbiota of the murine and human uGI tract can be distinguished from contamination by read count and taxonomic composition

**Figure 1.**
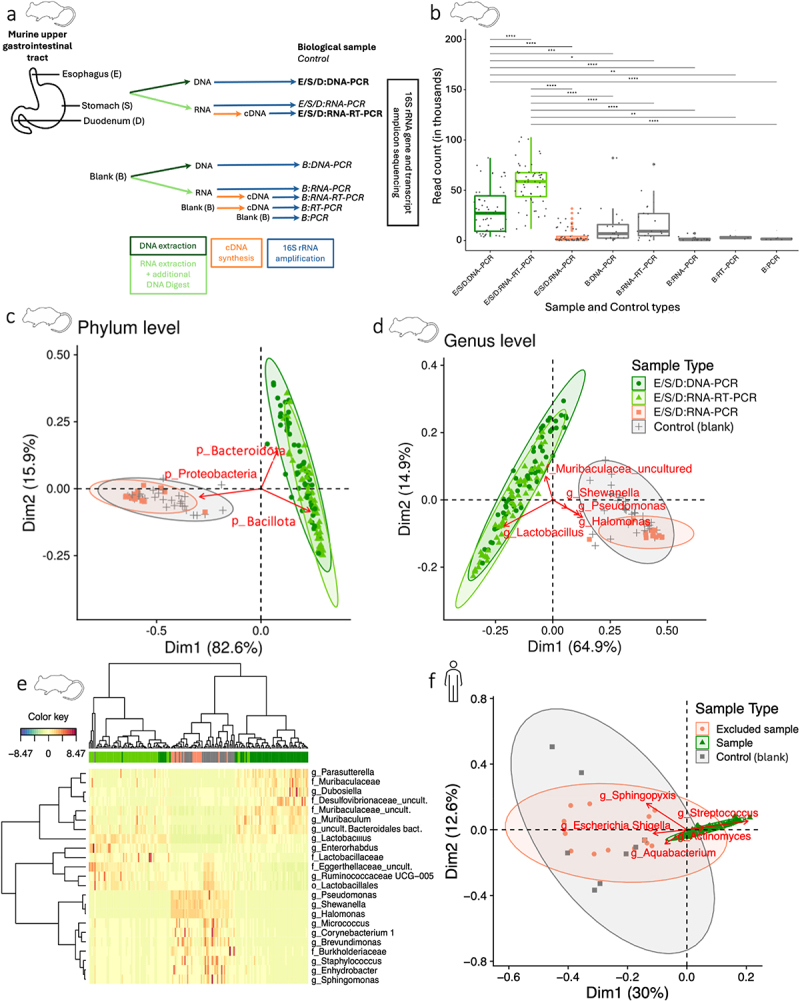
The DNA and RNA-based murine and human uGI tract microbiota is distinguishable from contamination.

To evaluate the influence of contamination on microbiota analysis of low-biomass samples of the uGI tract, mouse esophagus (E), stomach (S), and duodenum (D) samples were processed together with an extensive set of controls (Figure 1a). These controls were selected to determine potential contamination introduced during the different steps of the sample processing and amplification protocol. Specifically, to assess contamination from the entire sample extraction and amplification process, blank (B) controls (extraction reagents only, see Methods) were processed in parallel with the biological samples for DNA extraction and 16S rRNA gene amplification (B:DNA-PCR) or RNA extraction, cDNA synthesis and 16S rRNA gene amplification (B:RNA-RT-PCR). Additional blank controls measured contamination only during the different amplification steps, i.e. during 16S rRNA gene amplification (B:PCR) or cDNA synthesis and 16S rRNA gene amplification (B:RT-PCR). Finally, to check if RNA isolates could be contaminated with DNA, 16S rRNA gene amplifications were carried out directly on RNA extracted from biological samples (E/S/D:RNA-PCR) and from blank controls (B:RNA-PCR). In total, six different types of controls were sequenced and analyzed in addition to the biological samples (Figure 1a).

(a) Protocol overview. Biological samples are labeled as E (esophagus), S (stomach), and D (duodenum). Blank extraction and amplification controls are labeled with B. (b) Amplicon sequencing produced significantly fewer reads per sample in controls (gray: extraction and amplification controls, red: DNA contamination control in RNA isolates) compared to biological samples (dark green: DNA, light green: RNA). E/S/D:DNA-PCR (*n* = 57), E/S/D:RNA-RT-PCR (*n* = 57), E/S/D:RNA-PCR (*n* = 57), B:DNA-PCR (*n* = 19), B:RNA-RT-PCR (*n* = 19), B:RNA-PCR (*n* = 19), B-RT-PCR (*n* = 3), B-PCR (*n* = 3); Pairwise Wilcoxon rank sum test with Benjamini-Hochberg correction (p-value: ns > 0.05; * < 0.05; ** < 0.01; *** < 0.001; **** < 0.0001). Taxonomic microbiota compositions at the phylum (c) and genus (d) levels, shown as PCA Biplots of clr-transformed relative abundances. Ellipses represent 95%-confidence intervals and arrows represent the most relevant taxa for the separation of sample types. E/S/D:DNA-PCR (*n* = 57), E/S/D:RNA-RT-PCR (*n* = 56), E/S/D:RNA-PCR (*n* = 16), controls (*n* = 32). (e) Heatmap of the 23 most discriminative taxa (rows) between controls and biological samples (columns), as identified by Partial Least Squares Discriminant Analysis. Control (*n* = 32), E/S/D:DNA-PCR (*n* = 57), E/S/D:RNA-PCR (*n* = 16), E/S/D:RNA-RT-PCR (*n* = 57). (f) Taxonomic compositional microbiota differences at the genus level between human biological samples and controls, based on a PCA Biplot of clr-transformed relative abundances as above. Excluded samples (*n* = 14); samples (*n* = 107); controls (*n* = 14).

Sequencing of all control sample types generated significantly fewer reads per sample compared to the biological samples (Figure 1b). Biological samples were also distinguishable from controls based on their taxonomic microbiota compositions. Principal Component Analysis (PCA) of centered log-ratio (clr)-transformed relative abundances was performed at the phylum and genus levels, which identified the bacterial taxa that most strongly separated biological samples from controls (Figure 1c,d). At the phylum level, biological samples were marked by high abundances of *Bacteroidota* and *Bacillota*, whereas controls were dominated by *Proteobacteria* (Figure 1c). At the genus level, biological samples were dominated by *Lactobacillus* and an uncultured genus of *Muribaculaceae*, while *Halomonas*, *Pseudomonas* and *Shewanella* were abundant in controls (Figure 1d), in line with previously reported gastric microbiota compositions^15^ and typical contaminants of microbiota analyses.^30,31^

Partial Least Squares Discriminant Analysis (PLS-DA) and sparse PLS-DA identified those taxa that were most discriminative for different sample types. Heatmap clustering based on the 23 most discriminative features of the first two principal components mostly separated (i) biological samples from controls, (ii) DNA from RNA-based microbiota profiles in biological samples, and (iii) DNA contamination in extracted RNA from other blank controls (Figure 1e), indicating distinct and only partially overlapping sources of contamination in metagenomes and metatranscriptomes. While DNA contaminations in metagenomes were characterized by high contributions of *Brevundimonas*, *Micrococcus*, *Burkholderiaceae*, *Corynebacterium 1*, *Sphingomonas* and *Enhydrobacter*, DNA contaminations in metatranscriptomes were dominated by *Pseudomonas*, *Shewanella* and *Halomonas* (Figure 1e, Supplementary Fig. S1). These contaminating taxa dominated controls (mean: > 75% cumulative relative abundance), but accounted for less than 2% (mean: 0.5% cumulative relative abundance) in all biological samples (Supplementary Fig. S2), which were retained for further analyses.

Next, to analyze the human uGI tract microbiota, oral, gastric and duodenal samples were collected from a cohort of 20 individuals with mild, persistent ( > 6 months) uGI symptoms suggestive of non-classical food intolerance but with no indication of overt GI pathologies (Supplementary Table S1). Study participants tested negative for IgE-confirmed food allergies, celiac disease, inflammatory bowel disease (IBD) and carbohydrate malabsorption and underwent routine diagnostic uGI endoscopy for sample collection. Subjects provided saliva and gastric and duodenal samples were collected during EGD by flushing the stomach and duodenum with sodium chloride solution and recollecting the fluid. Since gastric and duodenal flush samples were expected to be low in microbial biomass, the influence of contamination was assessed as described for the murine data by comparing taxonomic compositions of biological samples with blank controls (Figure 1f). As for the murine data, the microbiota compositions of most biological samples could be clearly distinguished from controls (Figure 1f). Based on microbiota compositions, 13 out of 60 samples (one saliva RNA sample, 4 gastric RNA samples, 1 gastric DNA sample, 4 duodenal RNA samples and 3 duodenal DNA samples), all of which were also characterized by low DNA and RNA yields after extraction (mean: 0.5 ng/µl in excluded vs. 9 ng/µl in non-excluded samples), were excluded from further analyses (Figure 1f, Supplementary Table S2).

In summary, these findings demonstrate the utility of extracting, processing, sequencing and analyzing an extensive set of controls to assess the extent and quality of project-specific contaminations in low-biomass microbiota samples and to identify and remove samples from the analysis which lack microbiota signals beyond those expected from contamination in empty controls. They also validate the applied protocol for the simultaneous extraction of DNA and RNA from different sample types of the human and murine uGI tract microbiota.

### The murine and human stomach microbiomes are compositionally more similar to the duodenum than to esophagus or saliva

**Figure 2.**
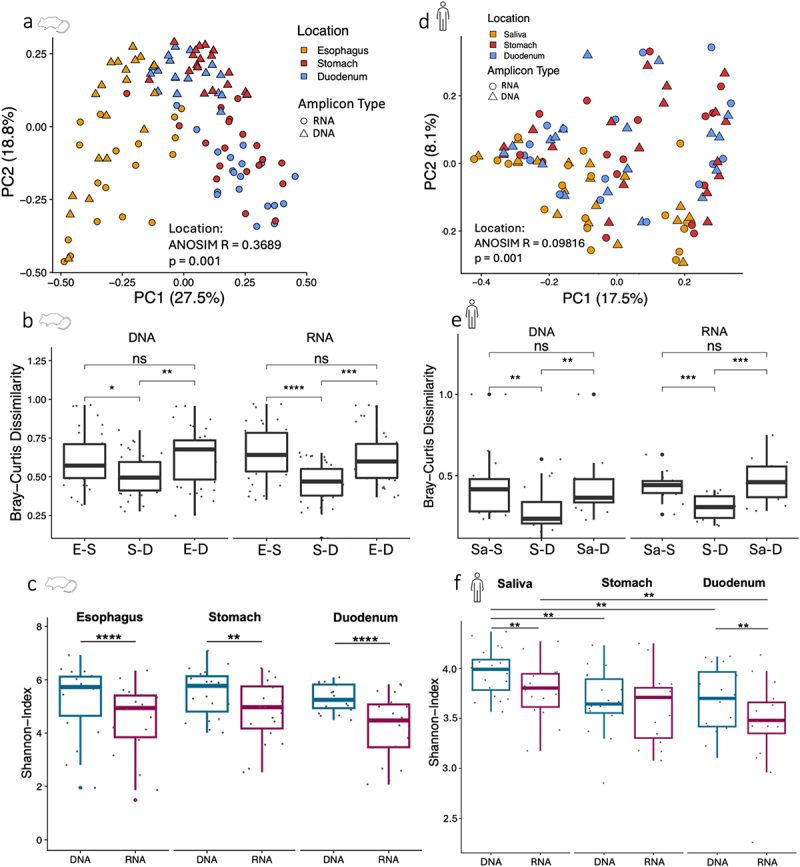
The murine and human stomach microbiomes are compositionally more similar to the duodenum than to esophagus or saliva.

In mice, microbiota diversity analysis revealed greater similarity of stomach and duodenum to each other than to the esophagus based on Bray-Curtis Dissimilarity (BCD), for both 16S rRNA gene and 16S rRNA transcript data (Figure 2a,b). Additionally, there was a separation of DNA and RNA along PC2. While within-sample diversity, measured by the Shannon Index, did not differ between locations, it was higher at each location in DNA compared to RNA (Figure 2c).

(a) Taxonomic compositional microbiota differences between locations and DNA and RNA, based on PCoA of Bray-Curtis Dissimilarity: ANOSIM *R* = 0.3689, *p* = 0.001, permutations = 999, and DNA/RNA: ANOSIM *R* = 0.3354, *p* = 0.001. DNA: esophagus (*n* = 18); stomach (*n* = 19); duodenum (*n* = 19); RNA: esophagus (*n* = 18); stomach (*n* = 19); duodenum (*n* = 19). (b) Boxplot of Bray-Curtis Dissimilarities (BCD) between locations, separately for DNA/RNA. BCD were calculated for each mouse between all three locations. DNA: esophagus-stomach (*n* = 19), stomach-duodenum (*n* = 19), esophagus-duodenum (*n* = 19); RNA: esophagus-stomach (*n* = 18), stomach-duodenum (*n* = 19), esophagus-duodenum (*n* = 18). (c) Microbiota diversity per sample (Shannon index) for esophagus, stomach and duodenum samples, separately for DNA/RNA. DNA: esophagus (*n* = 18); stomach (*n* = 19); duodenum (*n* = 19); RNA: esophagus (*n* = 18); stomach (*n* = 19); duodenum (*n* = 19). (d) PCoA of Bray-Curtis Dissimilarity for human samples; significant differences between locations: ANOSIM *R* = 0.09816, *p* = 0.001, permutations = 999, and DNA/RNA: ANOSIM *R* = 0.08838, *p* = 0.001. DNA: saliva (*n* = 20), stomach (*n* = 19); duodenum (*n* = 17); RNA: saliva (*n* = 18); stomach (*n* = 16); duodenum (*n* = 16). (e) Boxplot of Bray-Curtis Dissimilarities between locations, separated by DNA/RNA. BCD were calculated for each individual between all three locations. DNA: saliva-stomach (*n* = 19), stomach-duodenum (*n* = 17), saliva-duodenum (*n* = 17); RNA: saliva-stomach (*n* = 14), stomach-duodenum (*n* = 14), saliva-duodenum (*n* = 14). (f) Shannon index of DNA and RNA for saliva, stomach and duodenum samples. DNA: saliva (*n* = 20); stomach (*n* = 19); duodenum (*n* = 17); RNA: saliva (*n* = 18); stomach (*n* = 16); duodenum (*n* = 16). P-values based on Wilcoxon rank sum test with Benjamini–Hochberg correction (p-value: ns > 0.05; * < 0.05; ** < 0.01; *** < 0.001; **** < 0.0001).

In humans, the stomach microbiota was also compositionally more similar (BCD) to the duodenum microbiota than those of both locations were relative to saliva (Figure 2d,e). Microbiota dissimilarities between locations were generally smaller in humans compared to mice (mean BCDs humans (DNA): saliva-stomach: 0.44; stomach-duodenum: 0.28; saliva-duodenum: 0.41; mice (DNA): esophagus-stomach: 0.57; stomach-duodenum: 0.40; esophagus-duodenum: 0.63). Microbiota diversity (Shannon-Index) decreased from saliva to duodenum and at all sites was higher in DNA than RNA, except for human stomach samples (Figure 2f), supporting the notion that 16S rRNA transcript-based taxonomic profiling provides insight into the subfraction of the total uGI tract microbiota of mice and humans that is transcriptionally active.

### Distinct transcriptional activities characterize the murine and human uGI tract microbiomes

**Figure 3.**
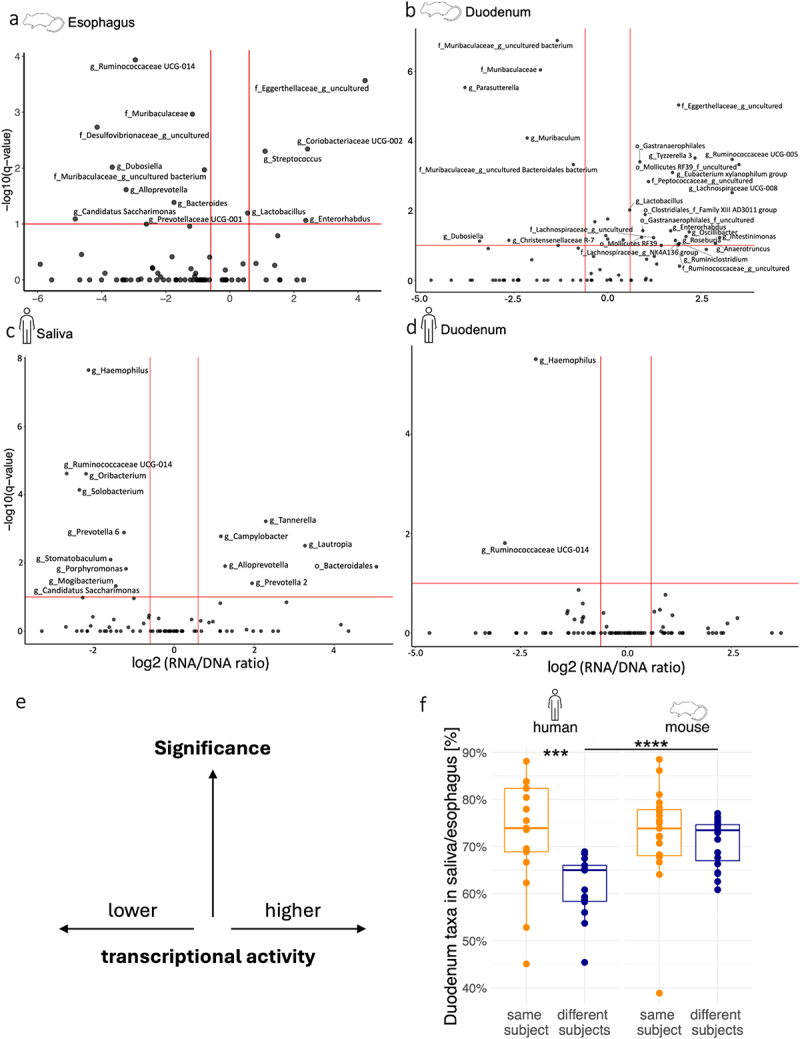
Transcriptional activity of murine and human uGI tract microbiota members.

Differences between total and transcriptionally active microbiota profiles at the murine and human uGI tract locations were determined with generalized linear mixed models (GLMM) (Supplementary Fig. S3). To highlight disproportionately active and inactive bacteria at the different uGI tract locations, the ratio of RNA-based to DNA-based relative abundances was calculated for individual bacterial taxa and plotted relative to the corrected p-values of the GLMM comparison of 16S rRNA gene and transcript-based taxonomic profiles (Figure 3e). In the murine esophagus, taxa with predicted high transcriptional activity included the genus *Streptococcus* (Figure 3a), in line with previous reports of this genus as a common murine oral and throat commensal.^32,33^ Transcriptionally inactive bacteria in the esophagus included several taxa described as intestinal bacteria (e.g. *Ruminococcaceae*, *Muribaculaceae*, *Bacteroides*). While four taxa showed signs of transcriptional activity in the esophagus, only two of those, an uncultured *Eggerthellaceae* genus and *Enterorhabdus*, retained activity in the stomach (Supplementary Fig. S4a). Instead, in gastric samples, an uncultured member of *Peptococcaceae*, *Peptostreptococcaceae* and *Lactobacillaceae* were enriched in RNA. In general, the highest number of active taxa was found in the duodenum (21 taxa) (Figure 3b), while stomach (5 taxa) and esophagus (4 taxa) harbored noticeably less disproportionately active bacteria. Most of the taxa with transcriptional activity signatures in the duodenum were not active in the esophagus and stomach (19/21 taxa) and have been described as strict anaerobes (16/21 taxa, Supplementary Table S3), suggesting that increased activities of these bacteria could be driven by lower oxygen concentrations in the duodenum compared to esophagus and stomach.^34^ Interestingly, the dominant genus in all murine locations, *Lactobacillus*, showed high transcriptional activity only in the esophagus and duodenum, indicating inhibition in the gastric milieu that is only apparent from the comparison of 16S rRNA gene and transcript, but not DNA-based alone, taxonomic microbiota profiles.

Taxa with significant differences in 16S rRNA gene versus transcript relative abundances in murine and human uGI tract samples are shown as volcano plots with log2-transformed DNA/RNA relative abundance ratios, calculated by dividing the mean relative abundances per taxon, and BH-adjusted q-values as determined by GLMMs. A positive log2 (RNA/DNA ratio) indicates high transcriptional activity, a negative log2 (RNA/DNA ratio) low transcriptional activity. Mice: a: esophagus, b: duodenum; humans: c: saliva; d: duodenum. Red vertical lines show BH-adjusted q-value >0.1; red horizontal lines show negative/positive log2 (RNA/DNA ratio) values of ±0.6. Taxa with a significantly higher relative abundance in 16S rRNA genes or transcripts (q < 0.1) are labeled. (e) Simplified schematic of volcano plots (a-d). (f) Fraction of detected genera in saliva (humans) or esophagus (mice) samples that is shared with duodenum samples from the same or different subjects. Human sample pairs: same (*n* = 17) and different (*n* = 17) subjects; mouse sample pairs: same (*n* = 19) and different (*n* = 19) subjects. Wilcoxon rank sum test with Benjamini-Hochberg correction (p-value: ns > 0.05; * < 0.05; ** <0.01; *** < 0.001; **** < 0.0001).

In human uGI tract samples, the largest number of transcriptionally active bacteria (6 genera) was identified in saliva (Figure 3c), including common oral commensals such as *Tannerella* and *Campylobacter*.^35^ While none of these taxa showed high transcriptional activity in the stomach, a member of the *Saccharimonadaceae* family, *Atopobium*, and *Fusobacterium*, an oral commensal with a suggested pathogenic potential in the colon,^36^ were found to be active in the gastric environment (Supplementary Fig. S4b). In contrast to mice, none of the detected bacterial taxa showed disproportionately high transcriptional activity in human duodenal samples (Figure 3d).

In coprophagic mice,^22,37^ the detection of putative intestinal bacteria with 16S rRNA transcript/gene-based signatures of transcriptional inactivity in esophageal samples described above (Figure 3a) could result from fecal microbiota ingestion. As coprophagy should also promote horizontal microbiota transfer between mice, shared taxa were determined between esophageal and duodenal samples from the same or different mice and compared to shared taxa between salivary and duodenal samples from the same or different human individuals (Figure 3f). As expected, human sample pairs from the same individual shared more taxa than saliva and duodenum samples from different individuals (shared in the same individual: 72.7% ± 2.7 s.e.m of all taxa; shared between different individuals: 61.8% ± 15.3 s.e.m of all taxa), in line with more frequent intra-individual compared to inter-individual microbiota transfer. However, esophageal and duodenal samples from different mice shared significantly more taxa than sample pairs from different humans (Figure 3f) and there was no difference in shared taxa fractions between sample pairs from the same or different mice (shared in the same mouse: 72.7% ± 2.3 of all taxa; shared between different mice: 70.9% ± 1.1 s.e.m), indicating horizontal microbiota exchange between mice.

### Microbiota organization and interdependencies at different locations of the human uGI tract

To characterize and compare microbiota community structures along the human uGI tract, taxonomic 16S rRNA gene-based microbiota profiles from saliva, stomach and duodenum were studied by correlation and cluster analysis. Hierarchical clustering identified between nine (duodenum) and 12 (stomach) groups of co-occurring genera per location, which were validated by silhouette analysis (Supplementary Fig. S5). Members of the duodenal microbiota showed the largest number of positive or negative correlations (58 correlations with *R* < 0.7 or *R* > 0.7), followed by saliva (49 correlations) and stomach (17 correlations). Similarly, fewer taxa were confidently assigned to clusters in the gastric compared to the salivary and duodenal microbiota (see silhouette widths; Supplementary Fig. S5), indicating that the stomach microbiota exhibits a less organized community structure than those of saliva and duodenum.

**Figure 4.**
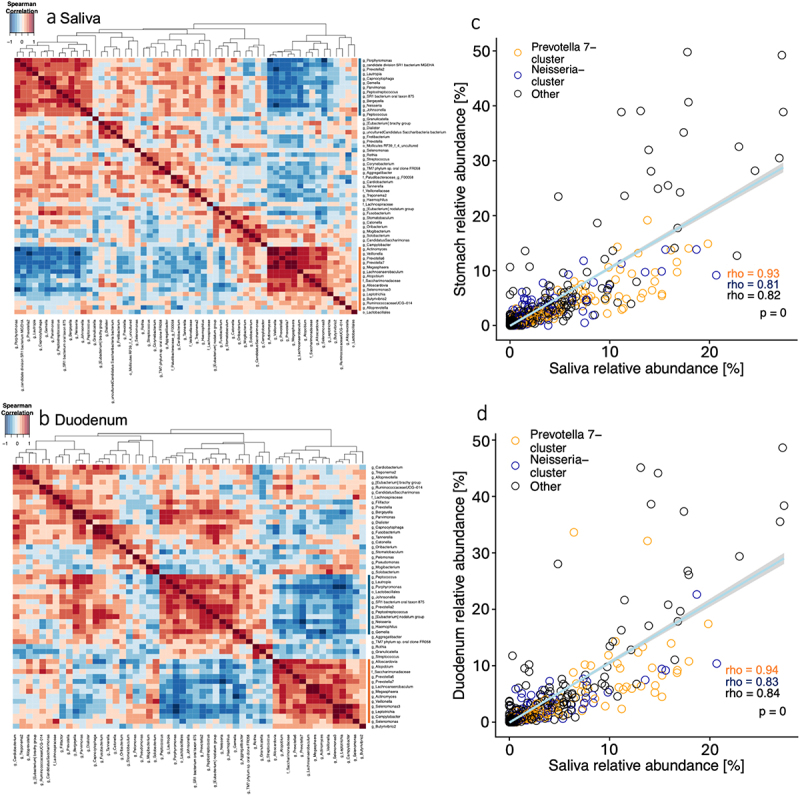
Microbiota organization and interdependencies at different locations of the human uGI tract.

The salivary microbiota was largely shaped by two distinct, negatively correlated clusters of 10 and 13 co-occurring taxa (silhouette width > 0), dominated by the genera *Prevotella 7* (mean relative abundance: 10.1% ± 1.1 s.e.m) or *Neisseria* (mean relative abundance: 6.5% ± 1.5 s.e.m), respectively (Figure 4a). The *Prevotella 7* cluster also included *Lachnoanaerobaculum*, *Megasphaera*, *Prevotella 6*, *Actinomyces*, *Veillonella*, *Alloscardovia*, *Atopobium, Saccharimonadaceae* and *Selenomonas 3*, with a mean cumulative relative abundance of 30% ± 2.7 s.e.m. The *Neisseria* cluster included *Porphyromonas, Bergeyella, Parvimonas, Prevotella 2, SR1 bacterium oral taxon 875, Peptostreptococcus, candidate division SR1 bacterium MGEHA, Capnocytophaga, Johnsonella, Gemella, Lautropia and Peptococcus*, with a mean cumulative relative abundance of 14% ± 1.7 s.e.m. Both clusters remained mostly intact in duodenal samples (Figure 4b), whereas only the *Prevotella 7*-dominated cluster could be detected in the stomach (Supplementary Fig. S6). No significant difference that would indicate disproportionately increased or decreased transcriptional activities was detected in the cumulative relative abundances of *Prevotella 7* or *Neisseria* cluster members between 16S rRNA gene and transcript data (Supplementary Fig. S7). Two overlapping clusters, including *Pseudomonas*, which was enriched in control samples (Figure 1), and other genera previously associated with contamination,^30,31^ were only detected in gastric (*Burkholderia*, *Pseudomonas*, *Pelomonas*, *Aquabacterium*) and duodenal (*Pseudomonas* and *Pelomonas*) samples, suggesting contamination as a stronger confounding influence at these uGI locations.

Spearman correlation matrix-based hierarchical clustering of the bacterial genera found in saliva (a) and duodenum (b), based on relative abundance profiles. The relative abundances were positively correlated between saliva and stomach (c) and saliva and duodenum (d) across all detected bacterial taxa (Spearman correlation).

Next, to assess microbiota interdependencies between the different human uGI locations, relative abundances were correlated between saliva, stomach and duodenum samples. Across all taxa, strong positive correlations were detected between saliva and stomach (rho = 0.82, Figure 4c) and between saliva and duodenum (rho = 0.84, Figure 4d). Positive correlations were even more pronounced for *Prevotella 7* cluster members (saliva-stomach: rho = 0.93, saliva-duodenum: rho = 0.94), indicating consistent contributions of this cluster to the microbiota of saliva, stomach and duodenum. Thus, salivary taxonomic microbiota profiles can provide indirect clues about the relative abundance of individual bacterial taxa in stomach and duodenum, especially with respect to members of the *Prevotella* 7-dominated cluster of co-occurring bacteria.

### Reproducible association of the Prevotella 7 microbiota type with reduced relative abundance of opportunistic pathogens

**Figure 5.**
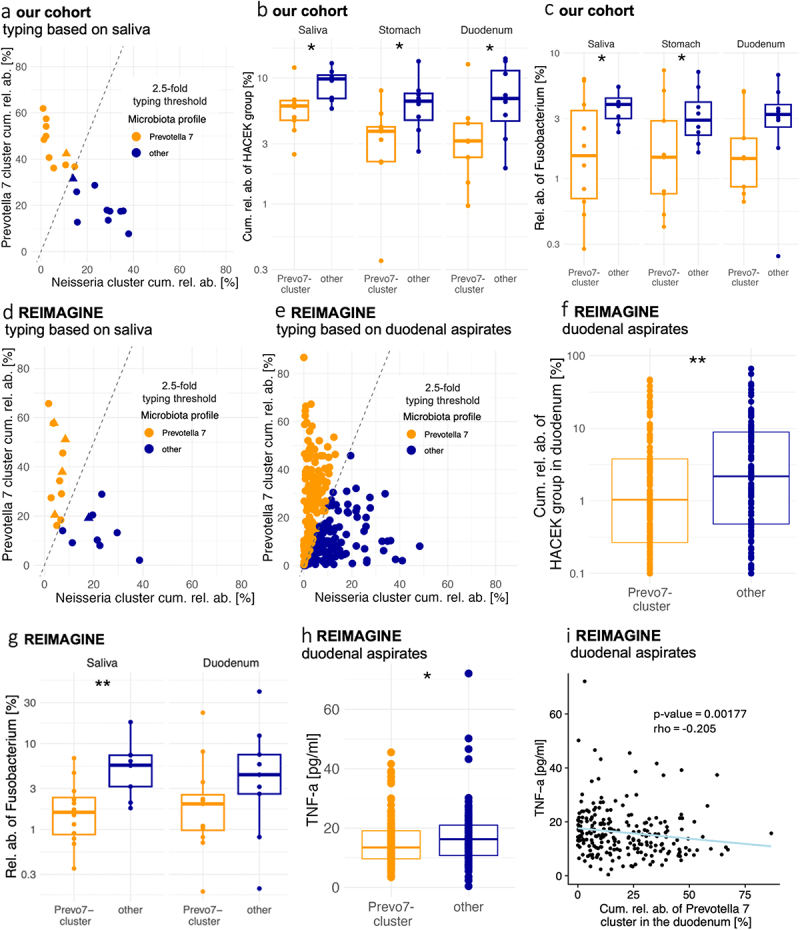
Assignment of individuals to cluster profiles and association with opportunistic pathogen burdens.

Across all subjects and locations, the cumulative relative abundance of *Prevotella 7* cluster members was about twice as high as that of the *Neisseria* cluster (27% ± 2.1 s.e.m. vs. 14% ± 1.4 s.e.m.) and both cumulative relative abundances were inversely correlated (Figure 4a,b, Supplementary Fig. S6). To determine whether the relationship between these two uGI microbiota clusters was a stratifying feature of our cohort and whether individuals could be assigned to uGI microbiota types based on cluster dominance, we assigned samples to the *Prevotella 7* microbiota profile if the cumulative relative abundance of *Prevotella 7* cluster members was at least 2.5-fold higher than that of *Neisseria* cluster members (Figure 5a). This threshold was selected based on the observation that most individuals harbored a *Prevotella 7*/*Neisseria* relative abundance ratio of either below 1.5 or above 3.0 (data not shown). Compared to other tested ratios (1.5/2.0/3.0-fold), the 2.5-fold threshold produced the strongest separation of individuals with respect to other microbiota features (Supplementary Fig. S8). Based on this typing scheme, 50% of the salivary microbiota profiles (10/20) were assigned to the *Prevotella 7* microbiota type and, except for two samples, individuals had consistent uGI microbiota types in saliva and duodenum (Figure 5a). Similar results were obtained based on 16S rRNA transcript relative abundance profiles (Supplementary Table S4). The *Prevotella 7*-dominated microbiota type thus appears to be an organizational uGI microbiota feature that in most individuals is shared between saliva and duodenum.

(a) Individuals can be categorized as either *Prevotella 7* cluster type or *other* that persist in saliva, stomach and duodenum, based on microbiota profiles. Plots show subjects with 2.5-fold the cumulative relative abundance of *Prevotella 7* (orange) assigned to cluster type *Prevotella 7*, else they get assigned to *other* (blue). Shapes indicate if cluster profile assignments remained consistent (round) or switched (triangle) in the duodenum (Prevo7 *n* = 10; other *n* = 10). (b) Cumulative relative abundances of bacteria belonging to the HACEK group for our cohort, separated by cluster type and location (saliva Prevo7 (*n* = 10) and other (*n* = 8) stomach Prevo7 (*n* = 9) and other (*n* = 8); duodenum Prevo7 (*n* = 9) and other (*n* = 8)). (c) Relative abundance of *Fusobacterium* by microbiota profile for saliva, stomach and duodenum for our cohort (saliva Prevo7 (*n* = 10) and other (*n* = 8); stomach Prevo7 (*n* = 9) and other (*n* = 8); duodenum Prevo7 (*n* = 9) and other (*n* = 8)). (d) Categorization into Prevo7 or *other* microbiota profile in the paired saliva-duodenum REIMAGINE samples (Prevo7 *n* = 12; other *n* = 9) and the duodenal aspirate samples (e) (Prevo7 *n* = 132; other *n* = 122). (f) Cumulative relative abundances of bacteria belonging to the HACEK group for the REIMAGINE duodenal aspirate samples (Prevo7 *n* = 132, other *n* = 122). (g) Relative abundance of *Fusobacterium* by microbiota profile for the paired saliva-duodenum samples of participants of the REIMAGINE study (saliva Prevo7 *n* = 12, other *n* = 9; duodenum Prevo7 *n* = 12, other *n* = 9). (h) Individuals from the REIMAGINE cohort that provided duodenal samples that were assigned to *other* microbiota profile had increased serum TNF-α levels (Prevo7 (*n* = 106), other (*n* = 96)). (i) In duodenal aspirates, the *Prevotella 7* cluster cumulative relative abundance correlates with TNF-a levels. Wilcoxon rank sum or t-test with Benjamini–Hochberg correction (p-value: ns > 0.05; * < 0.05; ** <0.01; *** < 0.001; **** < 0.0001).

As the oral microbiome can be a reservoir for pathogens that cause intestinal and extraintestinal infections,^38^ uGI microbiome profiles were queried for associations with the relative abundance of opportunistic pathogens. In individuals with the salivary *Prevotella 7* microbiota type, bacterial taxa from the HACEK group (*Haemophilus*, *Aggregatibacter*, *Cardiobacterium*, *Eikenella*, *Kingella*), a major cause of infective endocarditis and other infections,^39^ accounted for smaller microbiota fractions in all three locations (Figure 5b). In addition, subjects with the salivary *Prevotella 7* microbiota type were characterized by reduced relative abundance of the genus *Fusobacterium*, implicated in the development and progression of colorectal cancer and intestinal inflammation,^40^ in the saliva and the stomach (Figure 5c). In fact, despite the small cohort size, the cumulative relative abundance of *Prevotella 7* cluster members in saliva showed robust negative correlations with the duodenal relative abundance of both HACEK bacteria (*R* = −0.62, *p* = 0.008) and *Fusobacterium* (*R* = −0.55, *p* = 0.01), whereas only trends for positive correlations were observed between the salivary relative abundance of *Neisseria* cluster members and HACEK bacteria (*R* = 0.45, *p* = 0.07) or *Fusobacterium* (*R* = 0.44, *p* = 0.07) in the duodenum (Supplementary Fig. S9), suggesting that the salivary *Prevotella 7* microbiota type may be a better indicator for opportunistic pathogen burdens throughout the uGI tract.

Finally, the same *Prevotella 7* and *Neisseria* cluster-based typing scheme was applied to two datasets from the previously published, independent REIMAGINE study, a paired dataset of saliva and duodenum aspirate samples from 21 individuals and an overlapping dataset of duodenal aspirate samples from 254 individuals.^19,41^ As in the present cohort, similar percentages of individuals from the REIMAGINE cohorts were assigned to the *Prevotella 7* microbiota type based on saliva (12/21, 57%, Figure 5d) and duodenum samples (132/254, 52%, Figure 5e). In contrast to the present cohort (Supplementary Fig. S10a), a large fraction of duodenal aspirate samples (66/254, 26%) from the REIMAGINE cohort was characterized by low (< 10%) relative abundances of both the *Prevotella 7* and *Neisseria* cluster members (Figure 5e). As these samples were frequently dominated by a single bacterial genus (Supplementary Fig. S10b), including *Streptococcus* or members of the *Enterobacteriaceae* family (Supplementary Fig. S10c), additional separate uGI microbiota signatures may be associated with the clinically more severe disease backgrounds that characterize the REIMAGINE study participants (Supplementary Table S5).

Similar to the present study, HACEK relative abundance was reduced in duodenal aspirates from the REIMAGINE study assigned to the *Prevotella 7* microbiota type (Figure 5f) and showed the same trend in the paired saliva/duodenum dataset (Supplementary Fig. S11a). *Fusobacterium* relative abundance in saliva was also decreased in REIMAGINE study participants with the salivary *Prevotella 7* microbiota type (Figure 5g). Moreover, REIMAGINE individuals with the duodenal *Prevotella 7* microbiota type were characterized by significantly lower levels of serum TNF-α (Figure 5h), a proinflammatory cytokine that has been implicated in the pathogenesis of several chronic inflammatory disorders.^42^ And in the same individuals, TNF-α levels were negatively correlated (*R* = −0.205, *p* = 0.001) to the cumulative relative abundance of *Prevotella 7* cluster members in duodenal samples (Figure 5i).

In summary, these findings indicate a reproducible, consistent stratification of human cohorts based on *Prevotella 7* and *Neisseria*-dominated cluster profiles in saliva and duodenum, which is associated with uGI burdens of opportunistic pathogens and proinflammatory serum cytokine levels.

## Discussion

Low-microbial biomass microbiome samples from the uGI tract are particularly susceptible to confounding influences from contamination introduced during sample processing and amplification,^27,30^ which have not been carefully evaluated, particularly with respect to the analysis of 16S rRNA cDNA, which requires an additional reverse transcriptase amplification step. To carefully characterize and differentiate contaminating effects, establish robust DNA and RNA-based microbiota analysis protocols, and ensure the validity of the detected microbiota signals in biological samples, we compared murine samples to a comprehensive set of controls from all relevant nucleic acid isolation, amplification and sequencing steps. Previous reports have shown that insufficient contamination control in low-biomass microbiome studies can lead to misleading results, often reflecting reagent or environmental contaminants rather than true biological signal.^31,43^ Interestingly, contamination signals in our data varied between controls, reflecting different sources of contamination and highlighting the importance of project- and protocol-specific controls, rather than assuming nonspecific, consistent and comparable influences from the”kitome”^44^ across different projects. In our study, sequence data obtained from blank DNA and RNA extractions, blank cDNA synthesis reactions and blank PCRs were all dominated by *Brevundimonas*, *Micrococcus*, *Burkholderiaceae*, *Corynebacterium 1*, *Sphingomonas* and *Enhydrobacter*. In contrast, contaminating DNA in RNA extracts was characterized by increased *Pseudomonas*, *Shewanella* and *Halomonas* abundance, indicating that the combined protocol for DNA and RNA isolation introduced different types of DNA-based contaminations. The combined comparative analysis allowed us to distinguish biological samples from controls, based on sequencing read outputs and taxonomic microbiota compositions, attesting to the suitability of our protocol to generate reliable murine and human uGI tract microbiota data.

The murine and human uGI tract microbiomes share structural similarities, as previously shown.^15^ Here, we demonstrate increased compositional similarities between stomach and duodenum compared to esophagus and stomach (mouse) or saliva and stomach (human) for both hosts, consistent with a gastric microbiota disruption or reorganization within the uGI tract continuum.^15,45^ All locations were dominated by members of the Gram-positive order *Lactobacillales*, albeit from the genera *Streptococcus* in humans and *Lactobacillus* in mice, consistent with previous findings.^46^ Microbiomes from all murine and human GI locations were further characterized by reduced microbiota diversity in 16S rRNA transcript compared to gene amplicon sequence data, indicating that only a subset of bacteria detected in metagenomic DNA exhibits high transcriptional activity. Compared to humans, mice harbored more transcriptionally inactive bacterial taxa in the esophagus and more transcriptionally active taxa in the duodenum and most of these taxa belonged to species typically described as intestinal, anaerobic bacteria. Individual mice also shared more bacterial taxa with other mice, indicating horizontal microbiome transfer as a result from coprophagic behavior,^37^ which would be expected to increase the uGI abundance of intestinal microbes and to allow intestinally adapted bacteria to readily resume transcriptional activity in the duodenum of mice. Microbiome exchange between mice may also be facilitated by the higher gastric pH compared to humans (~4 vs. ~1.5),^47^ as well as other anatomical and physiological differences,^48^ but metagenomics-based microbiota analyses with subspecies resolution^49^ may be needed to track microbial strain transfer between mice and along the GI tract of individual mice. Since excessive bacterial growth, together with GI symptoms, defines small intestinal bacterial overgrowth (SIBO) as a pathological condition in humans,^50^ it would be of interest to determine if increased transcriptional activities of gut microbes in the duodenum of asymptomatic mice from our study were accompanied by higher microbial densities. Altered taxonomic and functional microbiota compositions in the small intestine of patients with GI symptoms undergoing testing for SIBO, rather than increased bacterial densities, have previously been associated with functional GI disorders,^51^ which may be in line with our findings and warrants further studies.

The small intestine, where most of the dietary absorption and immune surveillance takes place, plays an outsize role for host–microbe interactions and adverse reactions to food.^16–18^ Yet, the small intestinal microbiota is difficult to sample and our understanding of its composition, organization and function is limited compared to the large intestine, which has been extensively studied based on fecal microbiota analysis.^34,52^ A better understanding of the principles that govern uGI microbiota organization and site-specific interdependencies could help identify and modulate undesirable microbiome traits associated with dysbiosis and disease throughout the entire GI tract. Here, we demonstrate strong positive correlations between the relative abundances of individual bacterial taxa in human saliva and duodenal flush aspirates, supporting the previously suggested^19^ general feasibility of predicting duodenal microbiome features from oral microbiome analysis. Hierarchical clustering of bacterial relative abundance profiles identified two groups of co-occurring bacterial taxa, dominated by the genera *Prevotella 7* or *Neisseria*, which were inversely correlated and whose relative abundance ratio could be used to assign individuals from ours and the REIMAGINE^19,41^ cohort to distinct uGI microbiota types. *Prevotella* and *Neisseria* have previously been described as abundant genera in the human oral microbiota^53^ and assigned to variable, inconsistent salivary microbiota types that only partially overlap with our classifications. Both genera have been alternatively combined in a single commensal “salivatype” associated with healthy individuals^54^ or, as part of separate microbiota types, linked to vegan diet (increased *Neisseria*/*Prevotella* ratio),^55,56^ obesity (increased *Prevotella*)^55,56^ and geography (increased *Prevotella* in Japanese and increased *Neisseria* in Korean individuals).^57^ The relative abundance of *Prevotella* in saliva has also been positively correlated to gastroesophageal reflux disease (GERD,^58^ pancreatitis,^59^ duodenal tumors^60^ and inflammatory bowel disease (IBD,^61^ suggesting an adverse role of oral *Prevotella* for human health that would appear to contradict the positive association of our *Prevotella 7-*dominated uGI microbiota type with reduced opportunistic pathogen burdens. Moreover, similar inconsistent and even contradictory observations have been made with respect to the health and disease-related functions of intestinal *Prevotella*.^62^ However, the *Prevotella*/*Alloprevotella* complex has been undergoing taxonomic expansions and reclassifications in recent years, mostly based on comparative genome and metagenome analyses,^63,64^ in order to better reflect phylogenetic and functional variations that existed within the former *Prevotella* genus,^63,64^ which included > 15 oral “*Prevotella*” species alone.^62^ While the taxonomy of the *Prevotella*/*Alloprevotella* complex is difficult to fully resolve based only on 16S rRNA gene amplicon sequence data, direct comparisons of the most abundant amplicon sequence variants (ASVs) from our *Prevotella 7* and *Neisseria* clusters (Figure 4) with the most recent SILVA database (v138.2) suggests that *Prevotella 7* represents the actual *Prevotella* genus, based on the current taxonomy, whereas *Prevotella 6*, which is also part of the *Prevotella 7* cluster represents the new genus *Segatella*, and *Alloprevotella*, which used to be designated *Prevotella* , retains the *Alloprevotella* genus assignment. *Prevotella 2*, which is part of the *Neisseria* cluster, represents the new genus *Hoylesella*. Our findings, which are based on two separate, independent cohorts, thus shed new light on the organization and distribution of different genera within the *Prevotella*/*Alloprevotella* complex along the uGI tract, which could help resolve inconsistent associations of the former “*Prevotella*” genus with distinct salivary microbiota types and health conditions, and enable new, refined oral microbiota diagnostics.

Based on our data the *Prevotella 7*-dominated uGI microbiota profile may be associated with favorable infectious and inflammatory risk profiles.

First, members of the alternative *Neisseria*-dominated uGI microbiota type have been directly implicated in oral, intestinal and extraintestinal pathologies, including *Porphyromonas gingivalis* (periodontitis, cardiovascular disease, Alzheimer’s disease, and rheumatoid arthritis^65^) and *Parvimonas micra* (colorectal cancer).^66^

Second, along the entire uGI tract, individuals with the salivary *Prevotella 7*-dominated microbiota type from ours and the REIMAGINE cohort harbored reduced relative abundances of HACEK bacteria (*Haemophilus*, *Aggregatibacter*, *Cardiobacterium*, *Eikenella*, *Kingella*,^39^ which are responsible for 1–3% of all infective endocarditis cases.^39^ Given ambiguous guidelines and ongoing debates among dentists over the need for antibiotic prophylaxis prior to invasive dental procedures in patients with predisposing cardiac conditions and an increased risk for infective endocarditis,^67^ our findings suggest new, noninvasive risk assessments opportunities to guide personalized, preventive interventions. By screening patients for *Neisseria* or other non-*Prevotella 7*-dominated salivary microbiota types, at-risk populations could be identified and selected for enhanced oral hygiene monitoring and prophylactic antibiotic treatments before undergoing invasive dental procedures.

Third, individuals from ours and the REIMAGINE cohort with the salivary *Prevotella 7*-dominated microbiota type were characterized by reduced relative abundance of *Fusobacterium*, a risk factor for colorectal,^68^ oral^69^ and breast cancer,^70^ which has been implicated in exacerbating tumor growth, chemoresistance and metastasis in colorectal cancer.^40^ Extraintestinal infections with oral commensals, including *F. nucleatum*, *P. gingivalis*, or HACEK bacteria, are generally thought to originate from the oropharynx.^5,71,72^ Considering the observed positive correlation between the relative abundances of these and other taxa in saliva and duodenum, the risk for extraintestinal translocation of these opportunistic pathogens downstream of the oropharynx should also be explored.

Last, individuals from the REIMAGINE cohort with the duodenal *Prevotella 7* microbiota type were characterized by decreased serum levels of the pro-inflammatory cytokine TNF-α, and serum TNF-α concentrations were negatively correlated with the cumulative relative abundance of *Prevotella 7* cluster members in the duodenum. As a central cytokine to systemic inflammatory immune responses, TNF-α has become the target of biological therapies using neutralizing antibodies to treat chronic inflammatory and autoimmune pathologies, including rheumatoid arthritis, psoriatic arthritis, juvenile idiopathic arthritis, ankylosing spondylitis, psoriasis, Crohn’s disease and ulcerative colitis.^73–77^ The role of non-*Prevotella 7* uGI microbiota types for the diagnosis of systemic inflammation and the treatment of chronic inflammatory and autoimmune diseases with TNF inhibitors should therefore be further explored.

Together, our findings suggest that salivary microbiota analysis may be suitable for personalized uGI microbiota stratification and infectious and inflammatory risk assessment. The accessibility and non-invasive nature of saliva sampling and microbiota analysis makes this approach attractive for routine screening and longitudinal monitoring, which could be readily integrated into both clinical workflows and microbiome research settings. Future studies should validate the potential of personalized, predictive patient stratification models to identify patients at risk for uGI-associated infectious and inflammatory disease conditions.

The present study has several limitations. While the reported murine and human uGI characterizations are based on relatively small animal sample sets and patient cohorts, the validation of our findings in an independent, previously published patient cohort strengthens the reproducibility and generalizability of our key findings. Amplicon sequencing-based taxonomic microbiota analysis can be confounded by variable 16S rRNA gene copy numbers between bacterial taxa^78^ and differences in cDNA synthesis efficiency^79^ could have further affected RNA-based uGI microbiota profile comparisons. Although we have previously shown that human gastric biopsy and aspirate samples harbor microbiomes of comparable taxonomic compositions, at least in *H. pylori*-negative individuals,^15^ aspirate samples are characterized by increased microbial diversity^80^ and likely contain reduced relative abundances of mucosa-associated bacteria, limiting the comparability of murine tissue and human aspirate samples. While we standardized sampling volumes, different microbial densities and residual gastric and duodenal fluid volumes that were available from individual patients during flush sampling may have affected contamination levels and microbiota compositions. *Prevotella* abundance in saliva has been linked to diet,^81^ but detailed dietary information was not available for ours or the REIMAGINE cohort. However, major dietary confounders, such as recent dietary changes or adherence to elimination diets, were addressed in our exclusion criteria and the reproducibility of our findings in the REIMAGINE cohort suggest robust uGI traits despite potential dietary confounders. While 16S rRNA gene and transcript amplicon sequencing can only provide taxonomic uGI microbiota insights, metagenomic and metatranscriptomic analyses could also reveal functional and mechanistic information about uGI microbiota types and associations. However, shotgun sequencing approaches can be complicated by the “contamination” of samples with host DNA and RNA that can overwhelm microbiota signals, reinforcing the continuous relevance of microbiota-specific, PCR-based 16S rRNA gene amplification, especially for challenging, low-microbial biomass sample types, such as swabs and tissue samples.^82^ In any case, the presented methodology for contamination assessment through extensive controls could be similarly applied to 16S rRNA gene amplicon and metagenomics/metatranscriptomics-based microbiota studies. Finally, although individuals from our cohort presented with relatively mild clinical symptoms and no overt inflammatory, structural, or neoplastic GI disease, practical and ethical hurdles limited our ability to endoscopically sample and characterize the healthy uGI microbiota, which should be further studied.

## Conclusion

Our study exemplifies the application of carefully controlled protocols for the microbiota characterization of the murine and human uGI tract with 16S rRNA gene and transcript amplicon sequencing that can be adopted for other sequencing methods or low-microbial biomass settings in microbiome research. The identified total and transcriptionally active murine uGI microbiota profiles were consistent with horizontal microbiota transfer between mice as a consequence of coprophagy. Two clusters of co-occurring bacterial taxa, dominated by the genera *Prevotella 7* and *Neisseria*, were identified as conserved structural features of the uGI microbiota in separate cohorts that enabled uGI microbiota typing based on cluster dominance. Consistent microbiota assignments between paired saliva and duodenum samples and associations of the salivary *Prevotella 7* profile with reduced opportunistic pathogen burdens and an inflammation marker suggest potential saliva-based diagnostic applications, which should be studied further.

## Materials and methods

### Mouse and human samples

Whole-organ samples of esophagus, stomach and duodenum were collected from 19 healthy, adult, female Swiss wild type mice, which were co-housed at the Animal Care Facility of the University of Hohenheim, Stuttgart, Germany. Samples were obtained from excess mice of the facilities breeding program, which were exempt from ethics committee approval. Samples were immediately stored on ice and transported to the Department of Microbiome Research and Applied Bioinformatics at the University of Hohenheim where they were stored at −80°C in RNAlater until processing.

A cohort of 20 individuals (18 to 80 years) with suspected food intolerance was recruited as part of the INDICATE-FH cohort^83^ at the University Hospital Schleswig-Holstein (UKSH), Lübeck, Germany, with approval by the ethics committee of the University of Lübeck (approval number: AZ 19–233) and informed consent from all participants. As described elsewhere,^83^ patients were recruited from a gastroenterology outpatient setting and presented with persistent (> 6 months) upper GI symptoms suggestive of non-classical food intolerance. Inclusion criteria required no prior diagnosis of inflammatory, neoplastic, or structural GI disease, and a previous positive mucosal reaction to food triggers during confocal laser endomicroscopy (CLE) testing.^84^ Exclusion criteria were IgE-confirmed food allergies, recent elimination diets, recent use of antibiotics, PPIs, probiotics, prebiotics, or antiallergic medication, as well as known GI pathologies such as celiac disease, IBD, or carbohydrate malabsorption. Formal dietary intake assessments were not performed; however, participants were required to be in a stable dietary phase. See Supplementary Table S1 for further cohort details. Individuals donated saliva using standardized passive drool collection into sterile containers. A consistent sample volume of 1 ml saliva was then combined with 1 ml of RNAlater. Subjects were sampled for gastric and duodenal fluids via esophagogastroduodenoscopy (EGD), which was also used to rule out additional structural or biological uGI pathologies. EGD was performed at the endoscopy department of UKSH in Lübeck. During the EGD, 20 ml of sodium chloride solution was flushed onto the duodenal mucosa via the working channel. The fluid was then re-aspirated and 2 ml were transferred into sterile containers. After the sample collection, the tube was flushed again with sodium chloride solution into the duodenum to remove residual duodenal fluid contents before relocating the channel to the stomach, where the procedure was repeated. Saliva and gastric and duodenal flush samples were immediately stored at −80°C and shipped on dry ice to the Department of Microbiome and Applied Bioinformatics at the University of Hohenheim, Stuttgart, on dry ice, where they were stored at −80°C in RNAlater until processing.

For the REIMAGINE cohort, we accessed the publicly available species count data, as well as the cytokine and chemokine data (GM-CSF, IFN-γ, IL-10, IL-12p70, IL-13, IL-1β, IL-2, IL-4, IL-5, IL-6, IL-8, and MCP-1) (doi: http://10.22002/D1.1701).

### Parallel DNA/RNA extraction

For both murine and human samples, the protocol for simultaneous DNA/RNA extraction was based on the instructions of the ZymoBiomics DNA/RNA Miniprep kit (Zymo Research, Hilden, USA).

For DNA/RNA extraction from whole-organ tissue samples of mice, tubes with thawed tissue samples in RNAlater were vortexed (10 min/4°C) and centrifuged (15000 rcf/10 min/4°C). To harvest microbes from the luminal tissue surface, organs were cut open, expanded on a petri dish and rinsed twice with phosphate-buffered saline (PBS). To remove large food particles from the extraction, stomach samples were vortexed for 1 min in PBS and centrifuged at minimum speed (100 rcf) for 10 min. All samples were vortexed (1 min) and centrifuged again (15000 rcf/10 min/4°C) and the supernatant and visible floating tissue parts were discarded. The pellet was washed again with PBS and centrifuged (15000 rcf/10 min/4°C). This step was repeated for the pellets of stomach samples.

The human samples were centrifuged (15000 rcf/12 min/4°C). For both mouse and human samples, after centrifugation, supernatant was discarded and ZymoBiomics DNA/RNA lysis shield buffer was added (800 µl for mouse, 750 µl for human samples). Samples were transferred to MP-Lysis Matrix B tubes (MP Biomedical, MP Biomedicals, Eschwege, Germany) and cooled on ice before mechanical lysis by bead beating (5x 45 sec/6 m/s with a 5-min cool down on ice).

All following steps were executed according to the manual of the ZymoBiomics DNA/RNA Miniprep kit (Zymo Research, Hilden, USA) with the following modifications: To improve elimination of DNA in the RNA fraction, samples were treated with a prolonged DNAase I treatment at 37°C for 15 min. Before elution, DNA and RNA fractions were washed twice instead of once with the provided wash buffer. Eluted DNA was stored at −20°C and eluted RNA was stored at −80°C until further processing. Blank extraction controls were included for DNA (“B:DNA-PCR”) and RNA (“B:RNA-PCR”), which were treated the same as biological samples and only contained ZymoBIOMICS DNA/RNA lysis shield buffer.

### DNAse digest and cDNA synthesis

To remove DNA residues in RNA, RNA isolates were treated with an additional DNase digestion with the Turbo DNA-free kit following the Routine DNase treatment protocol by the manufacturer (Thermo Fisher Scientific Baltics UAB, Vilnius, Lithuania). Murine and human RNA isolates were reverse-transcribed into cDNA with random primers using the GoScript reverse transcriptase kit (Promega, Walldorf, Germany) following the manufacturer’s protocol. The DNA Clean and Concentrator Kit 5 (Zymo Research, Hilden, USA) was used according to the manufacturer’s protocol to clean up cDNA. Blank RNA extraction controls were included in the cDNA synthesis (“B:RNA-RT-PCR”), as well as blank cDNA synthesis controls without RNA template (“B:RT-PCR”).

### Library preparation and sequencing

For the mouse data, DNA, cDNA, original RNA samples and blank controls were used as templates for the targeted 16S rRNA gene sequence amplification of the hypervariable V4 region with Phusion High-Fidelity PCR Master Mix and Golay Barcoded Primers 515F and 806 R, including internal spacers of 0 to 7 bp length as previously described (79). For the normalization of PCR products, equimolar amounts were prepared using SequalPrep normalization plate kit 96 (Thermo Fisher Scientific, Waltham, USA). PCR products were pooled and concentrated to reduce the volume with the DNA Clean and Concentrator 5 Kit (Zymo Research, Hilden, USA). Sequencing Libraries were prepared with the NEBNext Ultra DNA library preparation kit (New England Biolabs, Ipswich, USA). Ultrapure, nuclease-free water was used as a blank sequencing control (B:PCR). Sequencing was conducted on an Illumina MiSeq Instrument using the MiSeq Reagent kit v3 with 600 cycles (Illumina, San Diego, USA) at the University of Hohenheim following the manufacturer’s recommendations.

For the human data, library preparation was performed using the Quick-16S NGS Library Prep Kit (Zymo Research, Hilden, USA) according to the manufacturer’s protocol. The final pooled (10 pM) and normalized (each sample to 30 ng DNA/RNA) library was sequenced on an Illumina MiSeq platform (Illumina Inc. San Diego, USA) using the Illumina MiSeq Reagent Kit V3-V4 (600-cycle).

The 16S rRNA gene amplicon sequence data is available at the European Nucleotide Archive (ENA; https://www.ebi.ac.uk/ena/) under the accession number PRJEB77929.

### Sequence processing

For the mouse data, initial preprocessing of raw sequencing data was performed with the open-source bioinformatics pipeline QIIME (Quantitative Insights Into Microbial Ecology.^85^ Raw sequencing reads were merged, demultiplexed and trimmed with Qiime1. All further steps were conducted in QIIME2.^86^ Open reference OTU clustering was performed to cluster the preprocessed sequences into operational taxonomic units (OTUs), defined by an identity threshold of the 16S rRNA gene sequence of 97%. For diversity analysis, all samples were rarefied to a sequencing depth of 3404 reads per sample.

For the human data, preprocessing of raw sequencing data was conducted using the open-source bioinformatics platform QIIME2. Primers and low-quality bases of demultiplexed sequencing reads were trimmed using the DADA2 workflow. Reads were filtered, denoised, merged and chimeras removed, retaining 75.54% of the total raw reads. Then, amplicon sequencing variants (ASV) were formed. For diversity analysis, sequences were rarefied to 2907 reads per sample. For differential abundance analysis in both datasets, rarefied data was classified at genus (L6) level using the Silva-132–99-nb-classifier.^87^ To account for recent reclassifications within the *Prevotella/Alloprevotella* complex, we conducted additional analyses of the five most abundant ASVs for each of the four relevant genera identified in our dataset. Taxonomic identities were reevaluated using the SILVA Alignment, Classification and Tree (ACT) web service (https://www.arb-silva.de/aligner/) against SILVA v138.2. Results confirmed that “*Prevotella 7*” represents the actual *Prevotella* genus; “*Prevotella 6*” within the same cluster, corresponds to *Segatella*; “*Prevotella 2*” found in the *Neisseria* cluster maps to *Hoylesella*; and *Alloprevotella* retains its current genus assignment. These clarifications are reflected in the discussion and taxon interpretations throughout the manuscript. The metadata files used in the QIIME and R workflows for mouse and human data are provided in Supplementary Table S8-S9.

### Statistical analysis

Data visualization and statistics were executed using R (v4.3.2). Normal distribution was evaluated using Anderson-Darling and Shapiro-Wilks tests. Non-normally distributed parameters were analyzed by non-parametric tests, such as (pairwise) Wilcoxon rank-sum test and normally distributed data was tested with parametric tests, such as t-test. Corrections for false discovery rates were performed with the Benjamini–Hochberg procedure. Unless indicated otherwise, boxplots show medians and corresponding 95% confidence intervals (CI) and significance thresholds with q > 0.05 ns, q < 0.05 *, q < 0.01 **, q < 0.001 ***, q < 0.001 ****. n-values represent the number of individuals and are listed in the figure legends for all statistical tests and significance thresholds.

For beta-diversity analysis, Bray-Curtis Dissimilarity was calculated between the relative abundances (rarefied to 2907 sequences) of pairs of samples. To visualize the multidimensional Bray-Curtis Dissimilarity Matrix, it was further reduced into a two-dimensional system and displayed in Principal Coordinate Analysis (PCoA) plots. For statistical testing of dissimilarities between sample groups, Analysis of Similarities (ANOSIM) was calculated. To compare microbial composition of biological samples and controls, Principal Component Analysis (PCA) of centered log ratio (clr)-transformed relative abundance data was conducted and displayed in a PCA Biplot. To enable comparison with controls, unrarefied data were used for this analysis. The PCA Biplot also integrates arrows to include factors (here: taxa) explaining the greatest variation between sample groups. To further characterize taxa most discriminative for a sample type, Partial Least Squares Discriminant Analysis (PLS-DA) and sparse PLS-DA (sPLS-DA) were conducted.^88^ For both datasets, associations between locations in the uGI tract and amplicon type (DNA/RNA) were determined with Generalized Linear Mixed Models (GLMMs). Phantom taxa, meaning such taxa with only a relative abundance > 1 for cDNA, but not DNA, were filtered out. Only taxa with a relative abundance of > 0.1% in at least 3 samples were considered, a pseudocount of 1 added for zero values, and the resulting relative abundances clr transformed. Location and amplicon type were included as fixed effects and individual mice/subjects as random effect and microbial taxa with significant differences between locations and/or amplicon types as determined based on estimated marginal means (EMMs) and a q-value < 0.1 based on FDR-corrected Tukey’s test were considered significant. Odds ratios with 95% CI, as well as marginal and conditional R2 were calculated for all significant models (Supplementary Table S6-S7).

Spearman’s correlation analyses were performed on relative abundance data. For location-intern correlations, taxa occurring in at least 5 samples were included. For hierarchical clustering, correlation coefficient matrices were transformed to distance matrices. Resulting dendrograms were cut at height 1, which resulted in the highest silhouette scores indicating best cluster assignment. Cluster validity was assessed by silhouette analysis, describing the degree of cohesion and separation of the clusters. We fitted a multinomial logistic regression model based on sex, age and disease classification and found no significant effect of these variables on microbiota profile assignment.

The QIIME commands and R code used for sequence processing, figure generation and statistical analysis are listed in Supplementary Table S10.

## Supplementary Material

Supplemental Material

SupplementaryTables_20250613.xlsx

## Data Availability

16S rRNA gene amplicon sequence data is available at the European Nucleotide Archive (ENA; https://www.ebi.ac.uk/ena/) under the accession number PRJEB77929. All additional information that is needed to reproduce the presented findings is included with the publication and its supplemental material.
