# Supplementary material for: Contamination-controlled upper gastrointestinal microbiota profiling reveals salivary-duodenal community types linked to opportunistic pathogen carriage and inflammation
Source: Gut Microbes. 2025 Aug 1;17(1):2539452. doi: 10.1080/19490976.2025.2539452 (PMC12320837; doi:10.1080/19490976.2025.2539452)
Supplement: Supplemental Material [file KGMI_A_2539452_SM1719.docx]

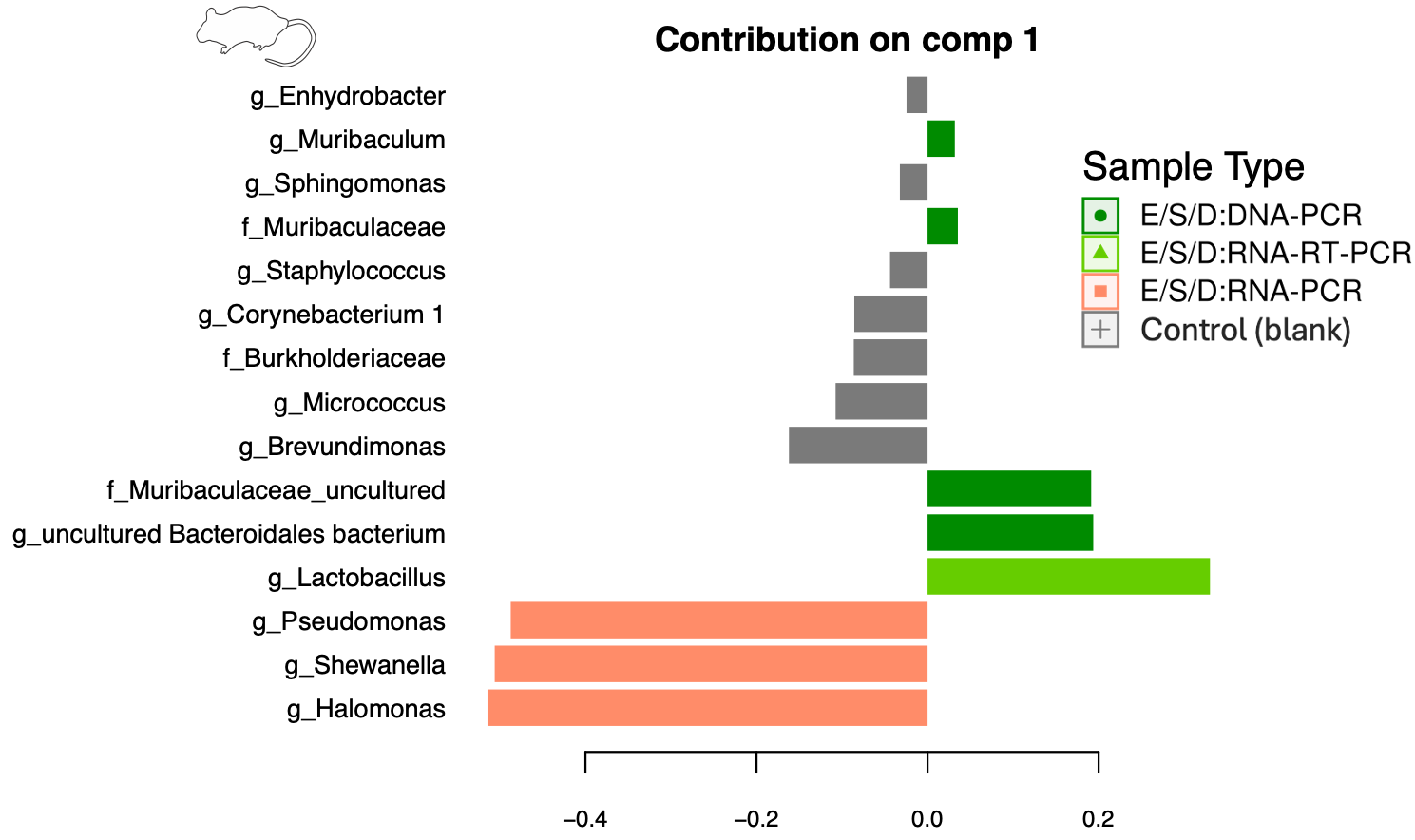


**Supplementary Figure 1:** Loading plot showing the 15 most discriminative taxa and their contributions to the separation of sample types along PC1 in Fig. 1d.

**
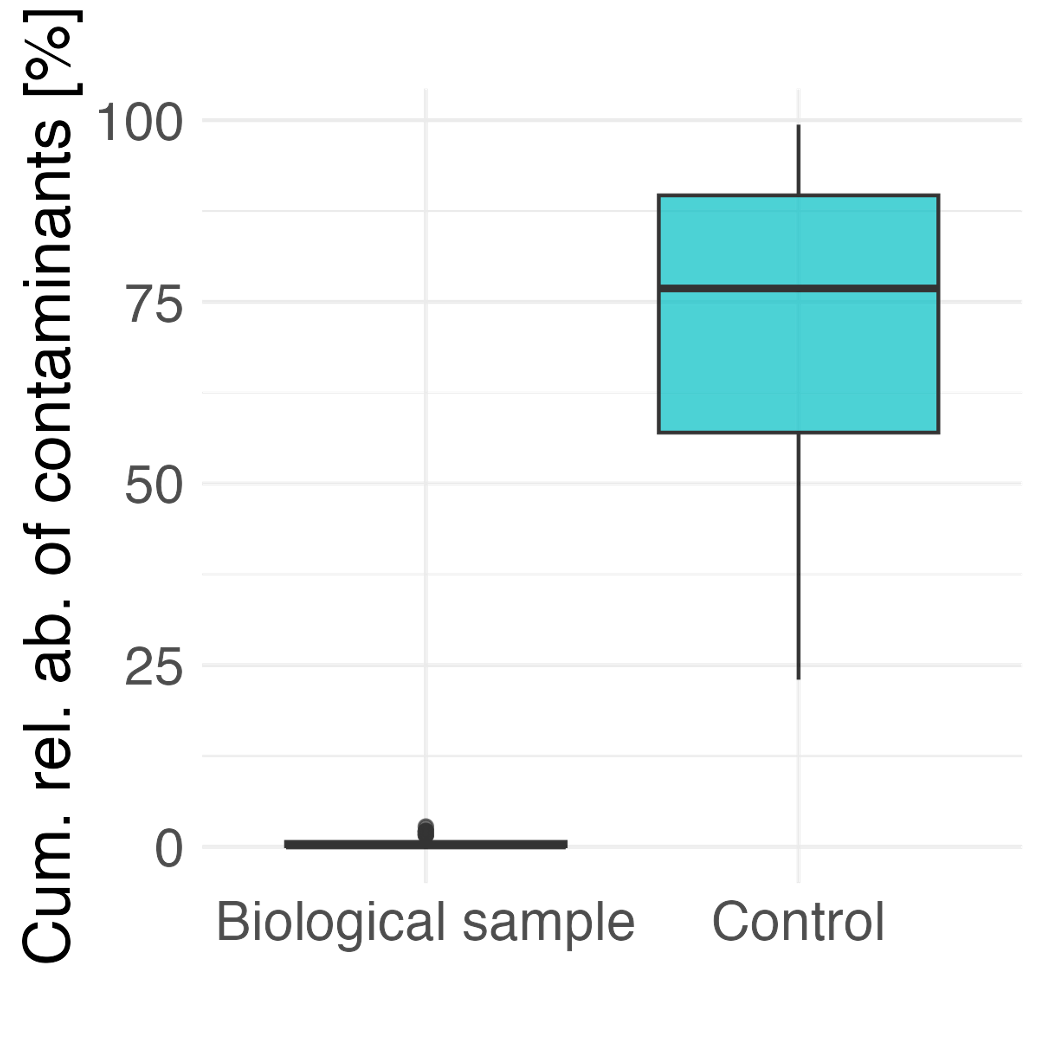
**

**Supplementary Figure 2:** The cumulative relative abundance of the 15 most discriminative taxa that separate controls from biological samples (Supplementary Fig. 1) is below 2% in all biological samples.

**
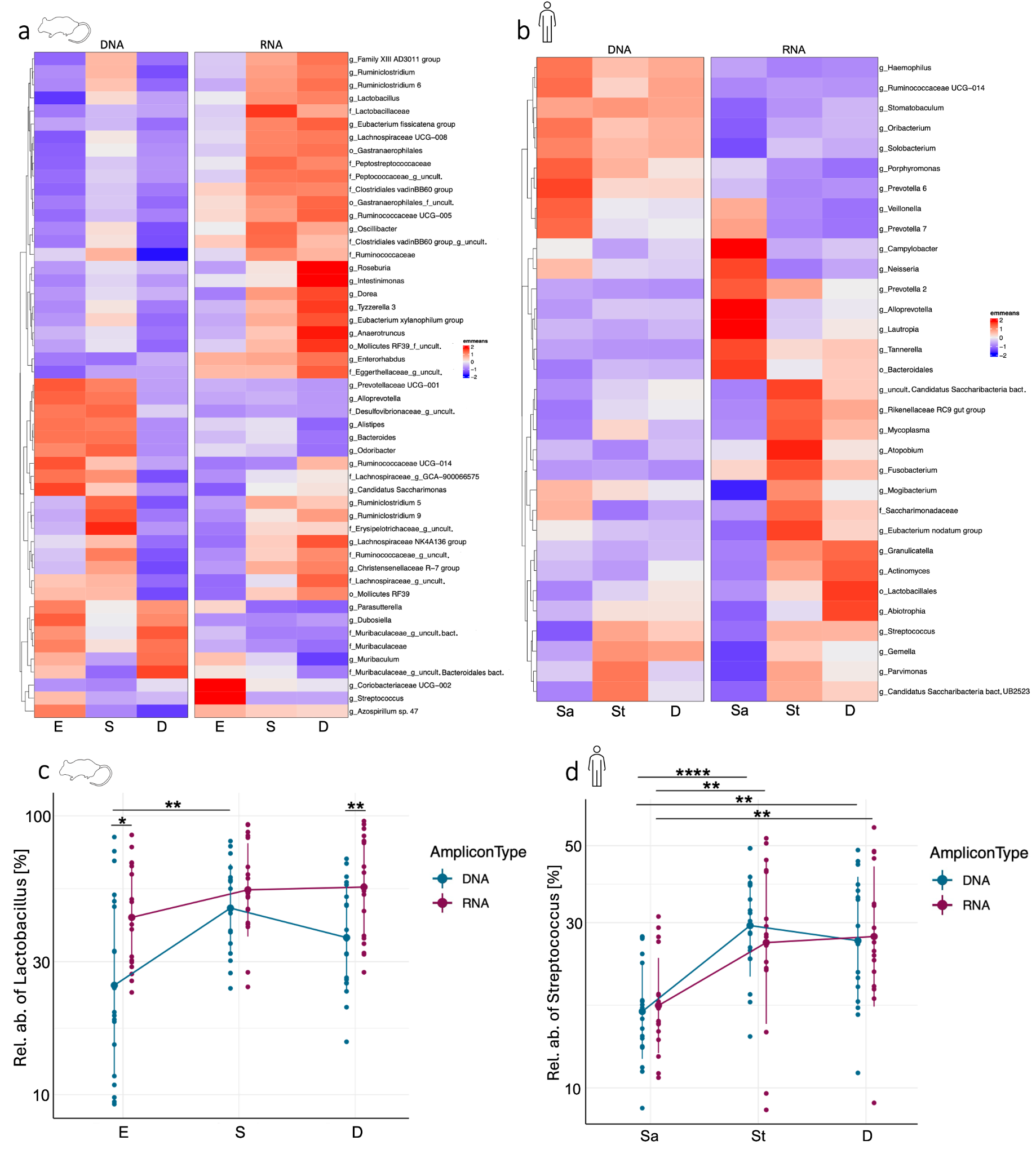
**

**Supplementary Figure 3: Bacterial taxa with changes in relative abundance between 16S rRNA gene or transcript microbiota profiles and uGI locations.** Heatmaps of all genera with significant (q < 0.1) differences in relative abundance based on Benjamini-Hochberg adjusted estimated marginal means (EMMs) as calculated by Generalized Linear Mixed Models (GLMM), scaled and centered over all locations and amplicon types for mice (a) and humans (b). Relative abundance of the most abundant genus across all uGI locations in (c) mice (*Lactobacillus*) and (d) humans (*Streptococcus*). Significant differences were determined with a GLMM. BH-adjusted EMMs (q-value: ns > 0.1, < 0.1 *, < 0.01 **, < 0.001 ***, < 0.0001 ****).

**
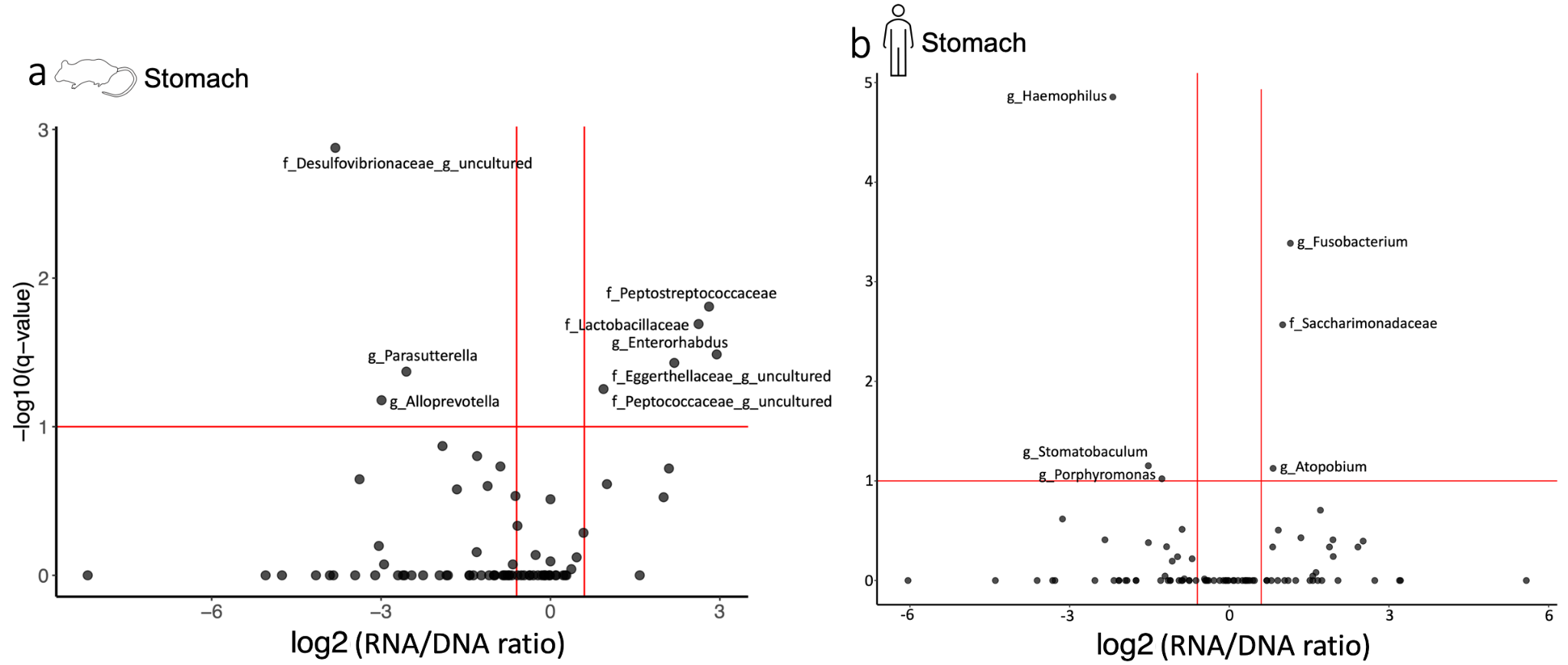
**

**Supplementary Figure 4: Transcriptional activity of the murine and human stomach microbiota.** Taxa with significant differences in 16S rRNA gene versus transcript relative abundances in murine (a) and human (b) uGI tract samples are shown as volcano plots with log2-transformed DNA/RNA relative abundance ratios, calculated by dividing mean relative abundances per taxon, and BH-adjusted q-values as determined by GLMMs**.** A positive log2 (RNA/DNA ratio) indicates high transcriptional activity, a negative log2 (RNA/DNA ratio) low transcriptional activity. Red vertical lines show BH-adjusted q-value >0.1; red horizontal lines show negative/positive log2 (RNA/DNA ratio) values of ±0.6. Taxa with a significantly higher relative abundance in 16S rRNA genes or transcripts (q < 0.1) are labeled.

**
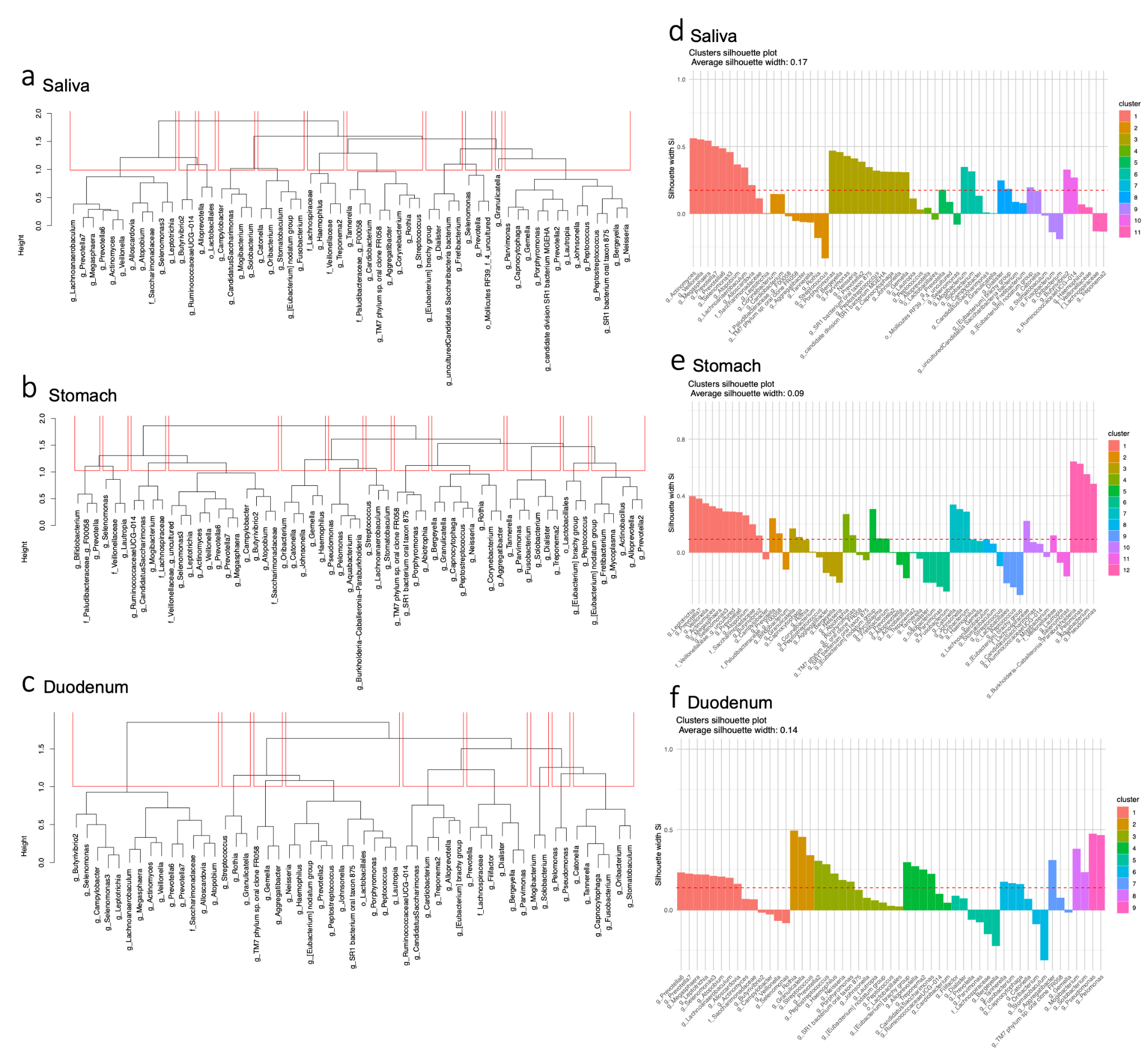
**

**Supplementary Figure 5: Cluster analysis of co-occurring taxa at different uGI locations.** Dendrogram of hierarchically clustered correlations of taxa in saliva (a), stomach (b) and duodenum (c), with red lines indicating clusters of taxa with a height <1.0. Silhouette analysis for hierarchical clustering for saliva (d), stomach (e) and duodenum (f). Taxa in each cluster are ordered by decreasing silhouette value. The silhouette value can range from −1 to 1. Large positive values indicate that clusters are distinct with greater intra-cluster similarity than between-cluster similarity. Negative silhouette values indicate inefficient cluster assignment. Colors show clusters forming with a height cutoff of 1.0. Dashed red lines represent the average silhouette score across all clusters.

**
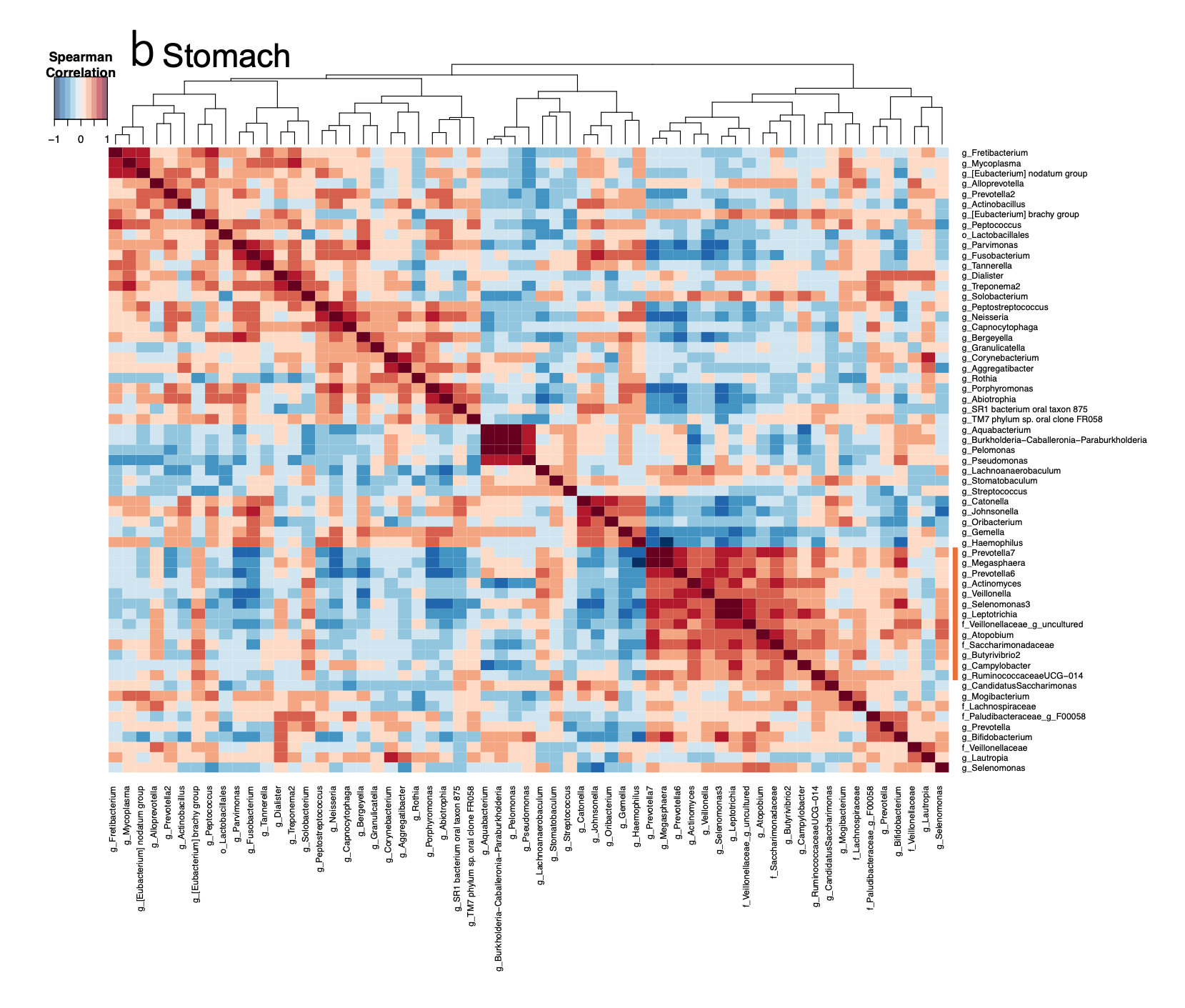
**

**Supplementary Figure 6: Co-occurring bacterial genera in the stomach.** Spearman correlation matrix-based hierarchical clustering of the bacterial genera found in stomach samples, based on DNA relative abundance profiles.


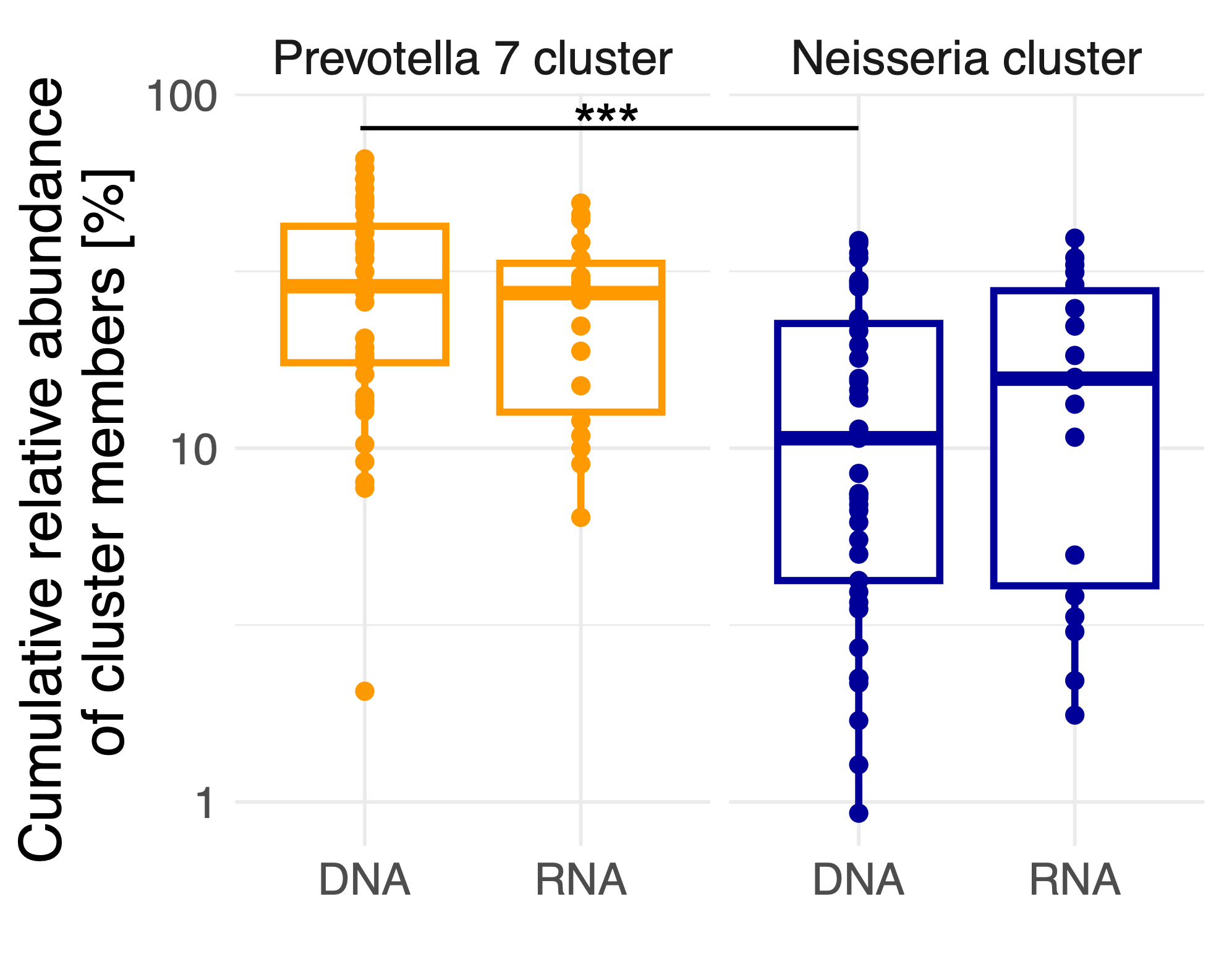


**Supplementary Figure 7:** **Cumulative relative abundances of *Prevotella 7* and *Neisseria* cluster members across all uGI locations shows no difference between DNA and RNA.** Wilcoxon rank sum test with Benjamini-Hochberg correction (p-value: ns > 0.05; * < 0.05; ** <0.01; *** < 0.001; **** < 0.0001).

**
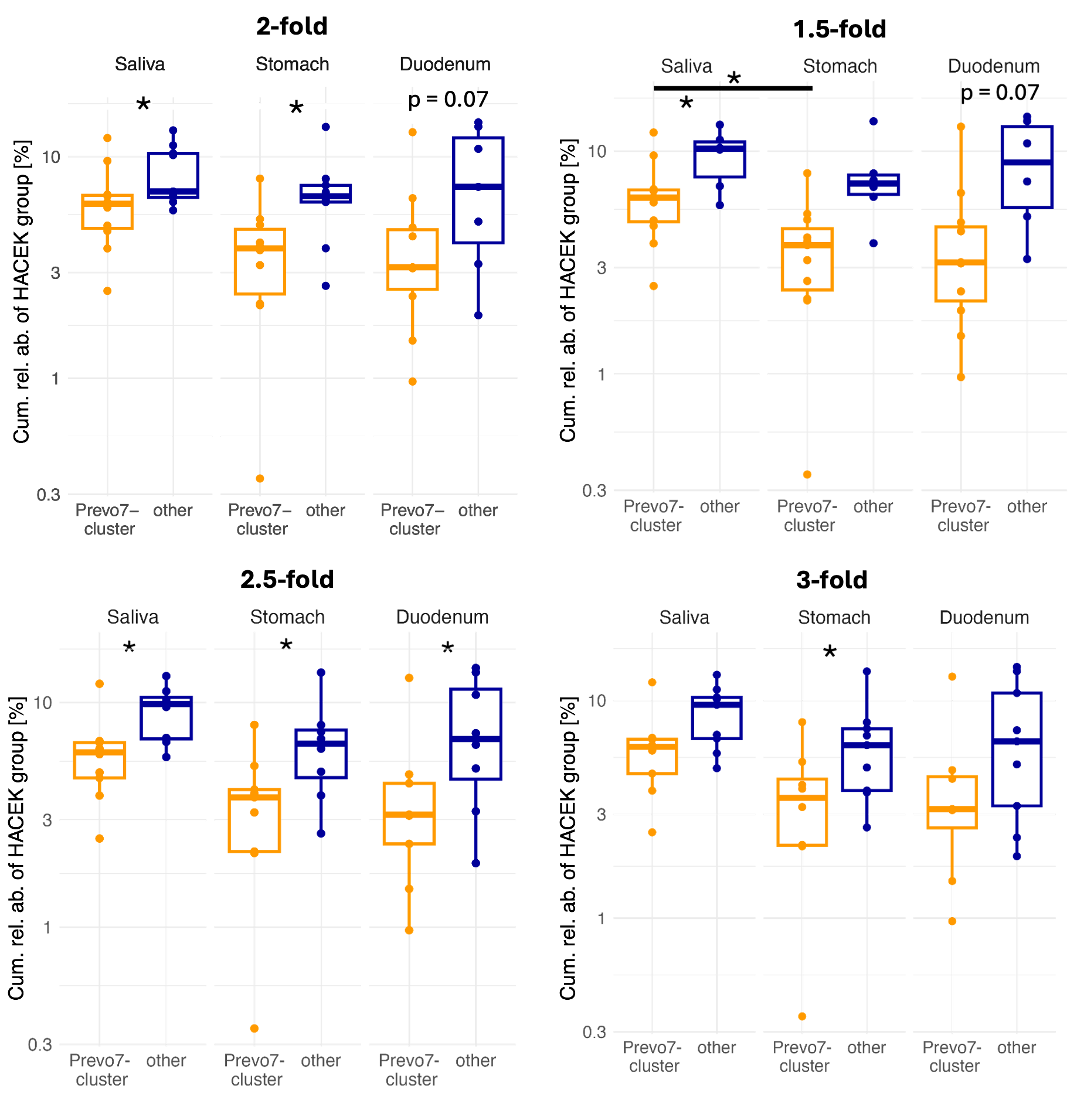
**

**Supplementary Figure 8:** Comparison of different *Prevotella 7*/*Neisseria* cluster cumulative relative abundance ratio thresholds on the difference in HACEK group relative abundance across locations.

**
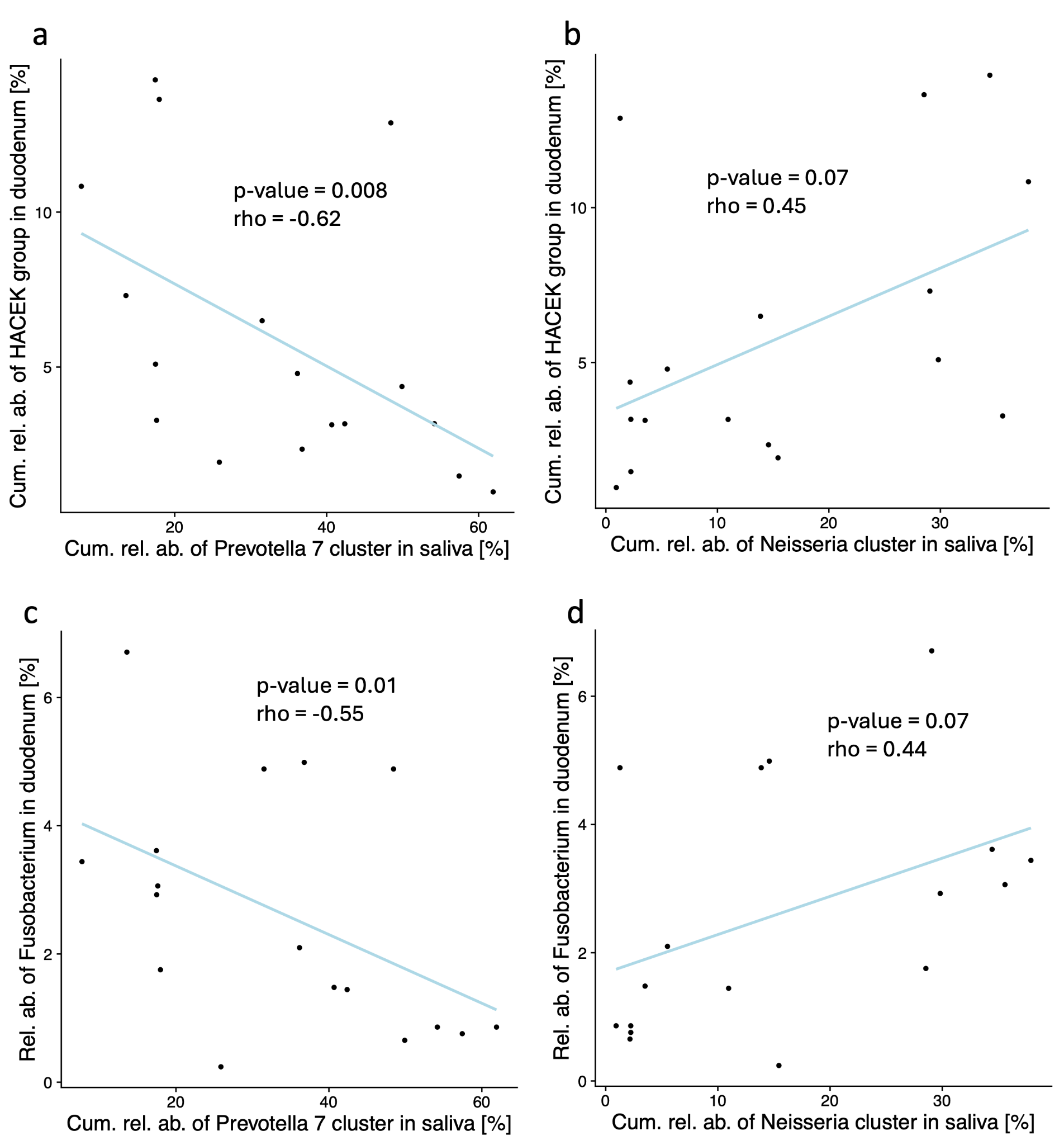
**

**Supplementary Figure 9: Negative association of the *Prevotella 7* microbiota type with HACEK bacteria and *Fusobacterium*.** Spearman correlation of *Prevotella 7* (a) and *Neisseria* (b) cluster member cumulative relative abundance in saliva with the cumulative relative abundance of bacteria from the HACEK group and *Fusobacterium* (c-d) in the duodenum.

**
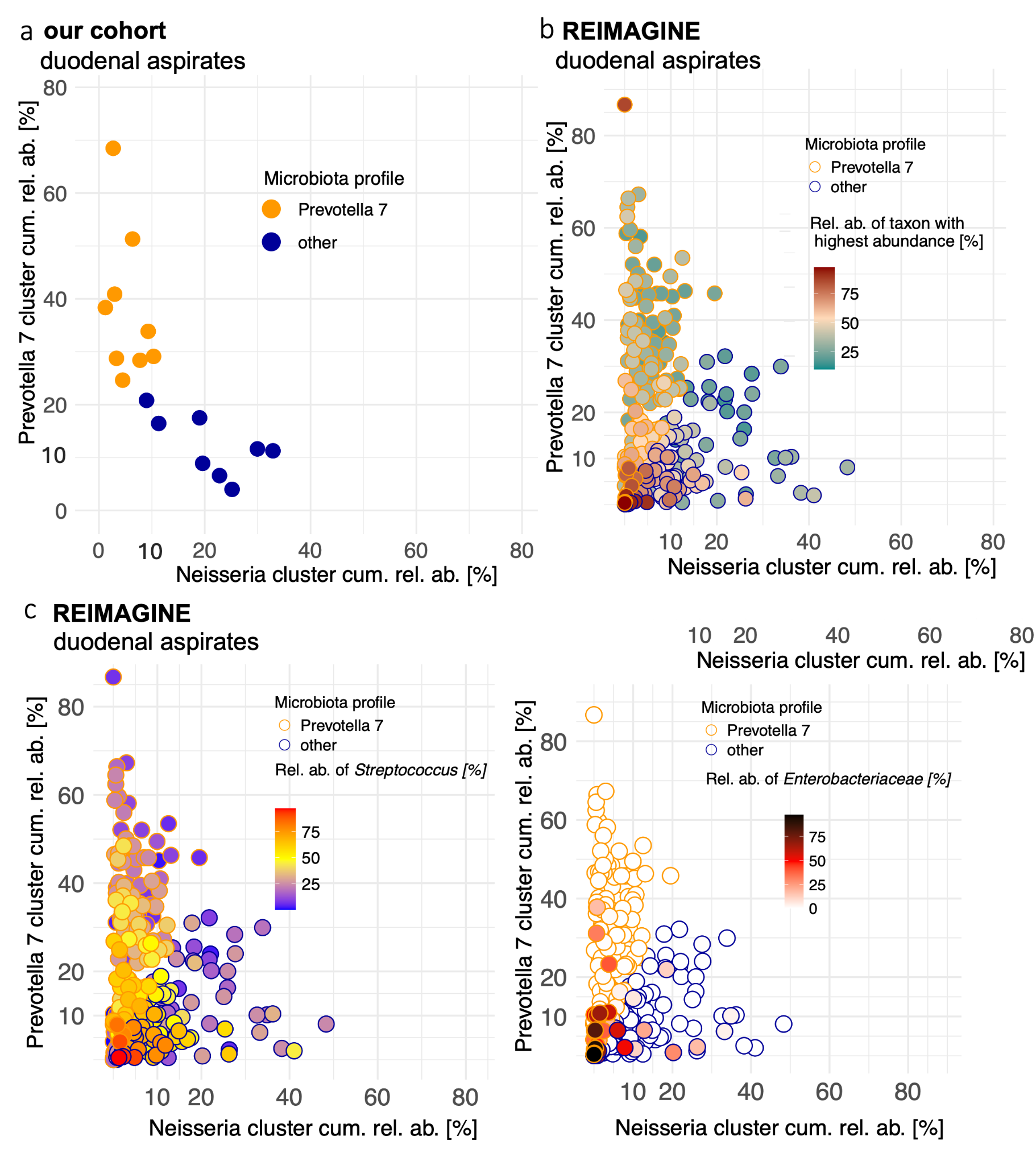
**

**Supplementary Figure 10: Frequent dominance of a single bacterial taxon in duodenal aspirates from the REIMAGINE cohort.** (a) All duodenal aspirate samples from the present cohort had cumulative relative abundances of the *Prevotella 7* and *Neisseria* clusters above 10%. Typing based on salivary samples. (n = 17; of those n = 8 are assigned to the *Prevotella 7* microbiota profile and n = 9 to *other*). (b) Duodenal samples from the REIMAGINE cohort frequently contained <10% cumulative relative abundance of both the *Prevotella 7* and *Neisseria* clusters but were instead dominated by a different single taxon. These dominating taxa included the genus *Streptococcus* or members of the family *Enterobacteriaceae* (c). Outline indicates microbiota type assignment, fill indicates cumulative relative abundance in %.

**
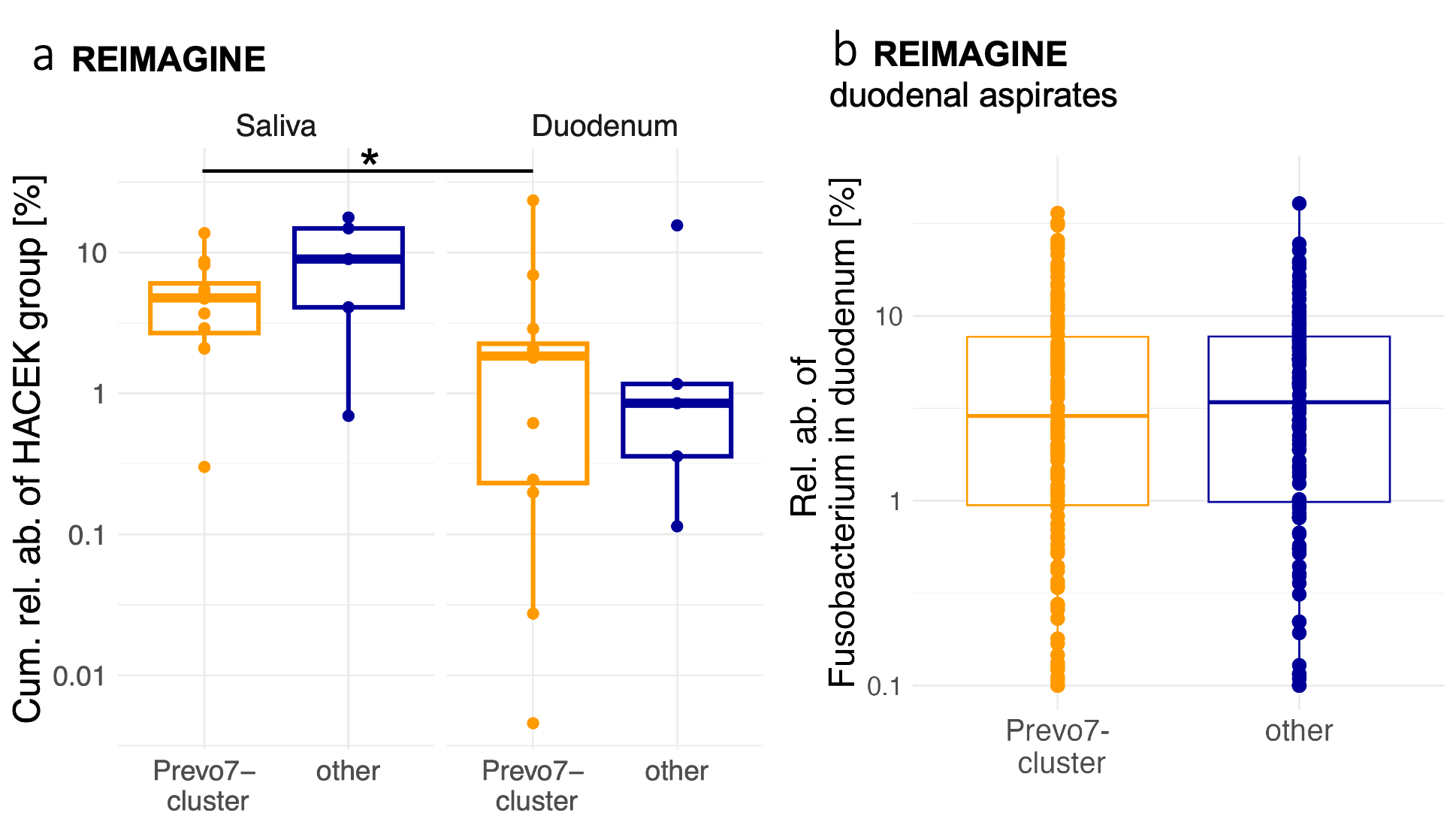
**

**Supplementary Figure 11: Opportunistic pathogen carriage in the REIMAGINE cohort.** (a) Cumulative relative abundance of bacteria from the HACEK group in paired saliva-duodenum samples of participants of the REIMAGINE study, typed based on salivary microbiota profiles (Prevo7 n = 12; other n = 5). (b) Relative abundance of *Fusobacterium* in the duodenal aspirate dataset of the REIMAGINE study (Prevo7 n = 132; other n = 122). Wilcoxon rank sum or t-test with Benjamini-Hochberg correction (p-value: ns > 0.05; * < 0.05; ** <0.01; *** < 0.001; **** < 0.0001).
